# LUBAC Suppresses IL-21-Induced Apoptosis in CD40-Activated Murine B Cells and Promotes Germinal Center B Cell Survival and the T-Dependent Antibody Response

**DOI:** 10.3389/fimmu.2021.658048

**Published:** 2021-04-19

**Authors:** Jingwei Wang, Tianbao Li, Hong Zan, Carlos E. Rivera, Hui Yan, Zhenming Xu

**Affiliations:** ^1^ Department of Microbiology, Immunology and Molecular Genetics, Joe R. and Teresa Lozano Long School of Medicine, University of Texas Health Science Center at San Antonio, San Antonio, TX, United States; ^2^ Division of Neonatology, Department of Pediatrics, The Second Xiangya Hospital, Central South University, Changsha, China; ^3^ Department of Molecular Medicine, Joe R. and Teresa Lozano Long School of Medicine, University of Texas Health Science Center at San Antonio, San Antonio, TX, United States

**Keywords:** affinity maturation, apoptosis, cFLIP, germinal center B cells, IL-21, linear ubiquitination, positive selection, SHARPIN

## Abstract

B cell activation by Tfh cells, i.e., through CD154 engagement of CD40 and IL-21, and survival within GCs are crucial for the T-dependent Ab response. LUBAC, composed of HOIP, SHARPIN, and HOIL-1, catalyzes linear ubiquitination (Linear M1-Ub) to mediate NF-κB activation and cell survival induced by TNF receptor superfamily members, which include CD40. As shown in this study, B cells expressing the *Sharpin* null mutation *cpdm* (*Sharpin^cpdm^*) could undergo proliferation, CSR, and SHM in response to immunization by a T-dependent Ag, but were defective in survival within GCs, enrichment of a mutation enhancing the BCR affinity, and production of specific Abs. *Sharpin^cpdm^* B cells stimulated *in vitro* with CD154 displayed normal proliferation and differentiation, marginally impaired NF-κB activation and survival, but markedly exacerbated death triggered by IL-21. While activating the mitochondria-dependent apoptosis pathway in both *Sharpin^+/+^* and *Sharpin^cpdm^* B cells, IL-21 induced *Sharpin^cpdm^* B cells to undergo sustained activation of caspase 9 and caspase 8 of the mitochondria-dependent and independent pathway, respectively, and ultimately caspase 3 in effecting apoptosis. These were associated with loss of the caspase 8 inhibitor cFLIP and reduction in cFLIP Linear M1-Ub, which interferes with cFLIP poly-ubiquitination at Lys48 and degradation. Finally, the viability of *Sharpin^cpdm^* B cells was rescued by caspase inhibitors but virtually abrogated – together with Linear M1-Ub and cFLIP levels – by a small molecule HOIP inhibitor. Thus, LUBAC controls the cFLIP expression and inhibits the effects of caspase 8 and IL-21-activated caspase 9, thereby suppressing apoptosis of CD40 and IL-21-activated B cells and promoting GC B cell survival.

## Introduction

B lymphocytes are central to humoral immunity, as they differentiate into “effector” cells that secrete class-switched (IgG, IgA, and IgE) and high-affinity mature antibodies (Abs) or “helper” cells that produce regulatory cytokines (1–3). During the Ab response, B cells expressing antigen (Ag)-specific B cell receptors (BCRs) are engaged by activated T follicular helper cells (Tfh) in secondary lymphoid organs, leading to their robust proliferation and differentiation in germinal centers (GCs), from which plasma cells emerge as long-lived Ab secreting cells (ASCs) and memory B cells are also generated to establish recallable immunity (3–5). Among the Tfh cell-expressed factors, CD154 (CD40 ligand, CD40L) engages CD40, a member of the TNF receptor superfamily, on target B cells and functions as a “primary” stimulus by inducing epigenetic modulation, activating transcription factor (e.g., NF-κB) and expanding the transcriptome to drive B cell proliferation and differentiation (2, 6). The sustained GC reaction requires IL-21, the hallmark cytokine of Tfh cells (7, 8), to boost B cell proliferation and induce plasma cell differentiation by upregulating BLIMP-1, the master transcription factor of plasma cells (9–12). IL-21 also synergizes with CD154 to induce AID, an activated B cell-restricted cytidine deaminase that introduces DNA lesions in the immunoglobulin (Ig) genes to initiate class switch DNA recombination (CSR) and somatic hypermutation (SHM), which underpin Ab class-switching (e.g., from IgM to different IgG isotypes) and affinity maturation, respectively (2, 13). Other CD4^+^ T helper cells, such as Th1 and Th2 cells, also participate in the GC reaction, e.g., through their hallmark cytokines. In particular, Th2 cytokine IL-4 synergizes with CD154 to activate NF-AT to enhance B cell proliferation as well as induce AID expression and IgH Iγ1-Cγ1 and Iε-Cε germline transcription to initiate CSR to IgG1 and IgE (2, 14).

CSR, most notably IL-4-dependent CSR to IgG1, is likely completed before the full development of IL-21^+^ Tfh cells and GCs (15, 16). Within the GC dark zone, class-switched B cells (and some IgM^+^ cells) acquire a high load of mutations in the V(D)J region DNA to alter the affinity of BCRs to the Ag. B cells bearing the BCRs of higher affinities are positively selected in the GC light zone and then shuttle back into the dark zone for new rounds of proliferation and SHM, an iterative process that eventually leads to affinity maturation (17). This can be manifested by the appearance – at the peak of the GC reaction – of several dominant BCR families whose members carry the signature mutation obtained by the founders. The positive selection of founder B cells and a subset of progenies in later rounds could be based on their superior survival in GCs, a notion consistent with that a high proportion of GC B cells are apoptotic and likely subclones outcompeted and failing to cross the survival threshold (18–20). Increasing evidence suggests that higher-affinity BCRs result in more efficient Ag uptake and MHC presentation to Tfh cells, which in turn endow GC B cells with a better survival capacity (21, 22). However, the stimuli that trigger the death of GC B cells and survival mechanisms employed by B cells are still poorly understood.

The linear ubiquitin chain assembly complex (LUBAC), which is composed of the HOIP catalytic subunit, SHARPIN structural subunit, and HOIL-1, is at the nexus of regulation of NF-κB activation and cell survival induced by TNF receptor superfamily members (23, 24). Prompted by this, we hypothesize that LUBAC plays an important role in CD40-mediated B cell proliferation, survival, and/or differentiation in response to immunization to T-dependent Ags. To test our hypothesis, we used a well-defined *in vitro* culture system to recapitulate the opposing impact of Tfh cell stimuli, i.e., induction of B-cell death by IL-21 and maintenance of survival by CD154, on B cells expressing the *cpdm* null mutation of *Sharpin* (*Sharpin^cpdm^*) and/or treated with HOIPIN-8, a recently discovered small molecule inhibitor of HOIP (25, 26). We also explored the molecular mechanisms underpinning IL-21-induced B cell apoptosis and the role of SHARPIN and LUBAC in regulating this process. We further addressed the B cell-intrinsic role of SHARPIN in specifically mediating the GC B cell survival and Ab responses to a T-dependent Ag *in vivo* by generating mice with B cell-specific expression of *Sharpin^cpdm^* (B-*Sharpin^cpdm^*) and mice in which *Sharpin^cpdm^* B cells directly competed against wildtype B cells within the same GC environment. Finally, we performed SHM analysis of over 20,000 BCR-encoding sequences to provide molecular evidence that B-cell SHARPIN promotes positive selection for high-affinity Abs.

## Materials and Methods

### Mice and Immunization

C57BL/6 (also CD45.2^+^, stock #000664), C57/CD45.1^+^ (B6.SJL-Ptprc^a^Pepc^b^/BoyJ, #002014), *Sharpin^cpdm/cpdm^* (*Sharpin^cpdm^*, #007599, CD45.2^+^) and μMT mice (#002288) were from the Jackson Laboratory. To generate mixed bone marrow chimera mice with B cell-specific *Sharpin^cpdm^* or *Sharpin^+/+^* genotype (B- *Sharpin^cpdm^* and B- *Sharpin^+/+^*, respectively), bone marrow cells from μMT mice (4x10^6^) and sex-matched *Sharpin^cpdm^*or *Sharpin^+/+^*littermate donor mice (10^6^) were mixed at the 80:20 ratio after red blood cell lysis and T cell depletion using a biotinylated anti-CD3 Ab and streptavidin-conjugated beads. Bone marrow cells were transplanted through intravenous (i.v.) injection into 8-week old C57 mice that had been γ-irradiated with a lethal dose (10 Gy) using a ^137^Cs source and recovered for 24 h in the presence of neomycin. Circulating leukocytes were monitored weekly by flow cytometry until full reconstitution of immune cells, typically after 8 weeks. To generate mixed bone marrow chimera mice carrying both CD45.2^+^ *Sharpin^cpdm^*and CD45.1^+^ *Sharpin^+/+^*hematopoietic lineage cells, 8-week old sex-matched CD45.2^+^ *Sharpin^cpdm^*and CD45.1^+^ C57 donor mice were co-housed for one week**before sacrificed to obtain bone marrow cells, which were mixed at the 50:50 ratio (2.5x10^6^ each) and injected i.v. into irradiated C57 recipient mice, as above. The immune cell reconstitution was verified by FACS.

All mice were maintained in a pathogen-free vivarium at the University of Texas Health Science Center at San Antonio (UTHSCSA). For immunization, mice were injected intraperitoneally (i.p.) with 100 μg of NP-CGG (in average 16 molecules of NP, 4-hydroxy-3-nitrophenyl acetyl, conjugated to one molecule of CGG, chicken γ-globulin; Biosearch Technologies) in the presence of 100 μl of alum (Imject^®^ Alum adjuvant, ThermoFisher) in the central-left abdomen area. Both male and female mice were used. All protocols were in accordance with the rules and regulations of the Institutional Animal Care and Use Committee (IACUC) of UTHSCSA.

### B Cell Isolation, Culture, and Stimulation

Mouse immune cells were isolated from single cell suspensions prepared from the spleen. Spleen cells were resuspended in ACK Lysis Buffer (Lonza) to lyse red blood cells. After quenching with RPMI 1640 medium supplemented with 10% FBS, 50 μM β-mercaptoethanol and 1x antibiotic-antimycotic mixture (Invitrogen) (RPMI-FBS), cells were resuspended in PBS for flow cytometry analysis or further preparation. To isolate B cells, splenocytes were subjected to negative selection (against CD43, CD4, CD8, CD11b, CD49b, CD90.2, Gr-1 or Ter-119) using EasySep™ Mouse B cell Isolation Kit (StemCell™ Technologies) following the manufacturer’s instructions, resulting in the preparation of more than 99% IgM^+^IgD^hi^ B cells. After pelleting, B cells were directly used for genomic DNA extraction for genotyping, RNA extraction, or resuspended in RPMI-FBS for stimulation. In genotyping experiments, non-B cells, i.e., those that bound the Ab cocktail and magnetic beads, were also subjected to genomic DNA extraction.

B cells were cultured (3.5x10^5^ cell/ml, 1 ml in a 48-well plate) in RPMI-FBS at 37°C and stimulated with CD154 (3 U/ml or as indicated), which was prepared as membrane fragments isolated from Sf21 insect cells infected by CD154-expressing baculovirus, to activate B cells for proliferation and differentiation, as described (6). The membrane fragments from non-infected Sf21 cells failed to activate B cells. IL-4 (5 ng/ml; R&D Systems) and IL-21 (50 ng/ml; R&D Systems) were added, as indicated. Other stimuli include an agonistic anti-CD40 Ab (clone FGK4.5; BioXcel; αCD40, 3 μg/ml or as indicated), TLR1/2 ligand Pam_3_CSK_4_ (100 ng/ml, Invivogen), TLR4 ligand lipid A (1 μg/ml, Invivogen), TLR7 ligand R-848 (30 ng/ml, Invivogen), TLR9 ligand ODN1826 (sequence 5’-TCCATGACGTTCCTGACGTT-3’) with a phosphorothioate backbone (CpG, 1 μM; Eurofins), F(ab’)2 of a goat anti-mouse μ chain Ab (anti-μ F(ab’) 2, 1 μg/ml; αIgM; Southern Biotech), which crosslinks IgM BCR, or anti-Igδ mAb (clone 11-26c conjugated to dextran, αIgD/dex, 100 ng/ml; Fina Biosolutions), which crosslinks IgD BCR (6). B cells were collected 24h later for RNA extraction or protein lysate preparation, or 96h later for FACS analysis of viability, proliferation, and expression of surface markers.

### Flow Cytometry and B Cell Proliferation Analysis 

To analyze B cells and other immune cells *ex vivo*, spleen or blood cells (2x10^6^) were first stained in Hank’s Buffered Salt Solution plus 0.1% BSA (HBSS-BSA) for 20 m with fluorophore-labeled mAbs to surface markers (**Supplementary Table 1**) in the presence of mAb Clone 2.4G2, which blocks FcγIII and FcγII receptors, and fixable viability dye (FVD) or 7–AAD without permeabilization. After washing, cells were resuspended in HBSS for FACS analysis. FACS data were analyzed by the FlowJo® software (BD).

To analyze B cell proliferation *in vivo*, mice were injected twice i.p. with 2 mg of bromodeoxyuridine (BrdU) in 200 μl PBS, with the first and second injection at 24 h and 1 h prior to sacrificing, respectively. Splenocyte (2x10^6^) were washed with BSA-HBSS and stained with Abs specific for surface markers in the presence of FVD. After washing, cells were resuspended in the BD Cytofix/Cytoperm™ buffer (250 μl) and incubated at 4**°**C for 20 m. After washing twice with the BD Perm/Wash™ buffer, cells were counted again and 10^6^ cells were resuspended in 100 μl of BD Cytofix/Cytoperm™ buffer for staining with fluorochrome-conjugated anti-BrdU mAb and/or 7–AAD for 30 m. After washing with BD Perm/Wash™ buffer, cells were analyzed by FACS. All data were analyzed by FlowJo^®^ (BD).

To analyze B cells stimulated *in vitro*, cells were harvested, stained with FVD or 7–AAD without permeabilization in HBSS-BSA for 20 m and analyzed by flow cytometry for expression of Igγ3, Igγ1 and Igγ2b expression (CSR to IgG3, IgG1, and IgG2b), CD138 (plasma cell marker) and other B cell surface molecules. To analyze B cell proliferation *in vitro*, CellTrace™ Yellow Cell Proliferation Kit (ThermoFisher) was used following the manufacturer's instructions with minor modifications. Briefly, 10x10^6^ purified naïve B cell were stained with CellTrace™ Yellow (10 μM, diluted from the 5 mM stock solution in 1 ml DPBS) for 5 m at 37°C with protection from light. After incubation, cells were pelleted by centrifugation for 5 m at 800g, washed with 10 ml RPMI-FBS before cultured for stimulation. Up to 10 cell divisions could be traced after 96 h of stimulation by CD154. B cells were harvested and analyzed by flow cytometry (Ex532nm, Em555/580nm).

### Cell Viability, Apoptosis, and Caspase Activity Analysis

For viability analysis, B cells were stained with 7–AAD, which enters the cell that had a compromised plasma membrane integrity, together with Abs specific surface marker in HBSS-BSA for 20 m, washed once, and resuspended in HBSS-BSA for FACS analysis. To analyze apoptosis and necrosis using 7–AAD and Annexin V, which binds to the phosphatidylserine that was located at the intracellular leaflet of the plasma membrane and exposed to Annexin V once “flipped” outside, B cells were stained with Abs specific for surface markers in HBSS-BSA on ice for 20 m. After washing, cells were resuspended in 100 μl Annexin V binding buffer (BioLegend) containing 7–AAD and 1:50 diluted FITC-conjugated Annexin V (BioLegend) at RT for 15 m. After staining, 400 μl of Annexin V binding buffer was added and cells were immediately analyzed by flow cytometry to analyze apoptotic (Annexin V^+^7–AAD^lo^) cells and necrotic (Annexin V^+^7–AAD^hi^) cells. The caspase activity in freshly harvested spleen B cells from immunized mice was detected using the CaspACE™ FITC-VAD-FMK (Promega), a FITC- conjugated analog of the pan caspase inhibitor Z-VAD-FMK that could diffuse into cells and irreversibly bind to all activated caspases. Splenocyte (10^6^) were cultured with CaspACE™ FITC-VAD-FMK (10 μM) in RPMI-FBS at 37°C for 15 m and then harvested for staining with Abs specific for surface markers in HBSS-BSA on ice for 20 m. After washing once and resuspended in HBSS-BSA, cells were analyzed by FACS. To analyze cleaved caspase 3 in B cells by intracellular staining, splenocyte (10^6^) were washed with BSA-HBSS and stained with Abs specific for surface markers in the presence of FVD. After washing, cells were fixed with the BD Cytofix/Cytoperm™ (200 μl) at 4°C for 20 m. After washing twice with BD Perm/Wash™ buffer, cells were stained in the same buffer with PE-conjugated Ab for cleaved caspase 3.

### Mitochondrial Membrane Potential Assay

Mitochondrial membrane potential (Δψm) was measured by the JC-1 Mitochondrial Membrane Potential Assay Kit (Abcam) following the manufacturer’s instructions. When the mitochondrial membrane potential is low, JC-1(tetraethylbenzimidazolylcarbocyanine iodide, a cationic dye) becomes a monomer that yields green fluorescence (JC-1 green) detectable in the FITC channel (530 nm Em) in flow cytometry. When the mitochondrial membrane potential is high, the dye accumulates into high concentrations in mitochondria, thereby aggregating and yielding red fluorescence (JC-1 red) detectable in the PE channel (590 nm Em). Stimulated B cells were washed with 1x dilution buffer and then stained with 10 μM JC-1 in 1x dilution buffer for 15 m at 37°C. Cells were washed once with 1x dilution buffer and stained for surface markers before flow cytometry analysis.

### ELISA and ELISPOT

Serum samples were collected at d 0, 7, and 14 after immunization. To analyze total serum IgM, IgG subclasses, and IgA tilter in homeostasis, samples collected at d 0 were diluted with PBS (pH 7.4) plus 0.05% (v/v) Tween-20 (PBST). Two-fold serially diluted samples and standards for each Ig isotypes were applied to 96-well plates coated with goat anti–IgM, anti–IgA or anti–IgG Abs (all 1 mg/ml, **Supplementary Table 1**) and incubated for 2h at 37 °C to capture IgM, IgA, and different IgG isotypes (IgG1, IgG2a, IgG2b, and IgG3) Abs. After washing with PBST, captured Igs were detected with biotinylated anti–IgM, –IgA, –IgG1, –IgG2a, –IgG2b and –IgG3 Abs (**Supplementary Table 1**) followed by reaction with horseradish peroxidase (HRP)-labeled streptavidin (Sigma-Aldrich), development with o-phenylenediamine and measurement of absorbance at 492 nm. Ig concentrations were determined using Prism^®^ (GraphPad). To analyze titers of high-affinity and total NP-specific Abs, plates were coated with NP_7_-BSA (7 NP molecules on one BSA molecule) and NP_34_-BSA, respectively. Captured Igs were detected with anti–IgM, –IgG1, –IgG2b, –IgG3, and –IgA Abs. Data are relative values based on end-point dilution factors.

For ELISPOT analysis of NP_7_-binding and total IgM^+^ and IgG1^+^ ASCs, Multi-Screen^®^ filter plates (Millipore) were activated with 35% ethanol, washed with PBS, and coated with anti–IgM, anti–IgG or NP_7_-BSA (all 5 μg/ml) in PBS. Single spleen or bone marrow cell suspensions were cultured at 250,000 cells/ml (in plates coated with NP_7_-BSA) or 125,000 cells/ml (in plates coated with anti–IgM or anti–IgG) in RPMI-FBS at 37°C for 16 h. After supernatants were removed, plates were incubated with biotinylated goat anti-mouse IgM or –IgG1 Ab, as indicated, for 2 h and, after washing, incubated with HRP-conjugated streptavidin. Plates were developed using the Vectastain AEC peroxidase substrate kit (Vector Laboratories). The stained area in each well was quantified using the CTL Immunospot software (Cellular Technology) and depicted as the number of spots for quantification.

### RNA Isolation, qRT-PCR and RNA-Seq

Total RNA was extracted from 5 x 10^6^ B cells using the RNeasy Mini Kit (Qiagen). First-strand complementary DNA (cDNA) was synthesized from equal amounts of total RNA (4 μg) using the SuperScript III System (Invitrogen) and an oligo-dT primer. cDNA was analyzed by qPCR using SYBR Green (Bio-Rad) and appropriate primers (**Supplementary Table 2**). PCR was performed in a CFX96™ Real-Time PCR System (Bio-Rad Laboratories) according to the following protocol: 95°C for 30 s, 40 cycles of 95°C for 10 s, 55°C for 30 s, 72°C for 30 s. Melting curve analysis was performed at 72°C–95°C. The ΔΔCt method was used to analyze transcript levels and data were normalized to the expression of *Cd79b*, which encodes BCR Igβ, as constitutively expressed in B cells.

For RNA-Seq, after RNA integrity was verified using an Agilent Bioanalyzer 2100™ (Agilent), RNA was processed using an Illumina TruSeq RNA sample prep kit v2 (Illumina). Clusters were generated using TruSeq Single-Read Cluster Gen. Kit v3-cBot-HS on an Illumina cBot Cluster Generation Station. After quality control procedures, individual RNA-Seq libraries were pooled based on their respective 6-bp index portion of the TruSeq adapters and sequenced at 50 bp/sequence using an Illumina HiSeq 3000 sequencer. The resulting reads, typically 16 million reads per sample, were checked by assurance (QA) pipeline and initial genome alignment (Alignment). De-multiplexing with CASAVA was employed to generate a Fastq file for each sample. After removing the adaptor and poor-quality reads using Trim Galore, all sequencing reads were aligned with the (GRCm38/mm10) reference genome by using HISAT2 with default settings, yielding Bam files, which were further processed using HTSeq-count to obtain counts for each gene. RNA expression levels were determined using GENCODE annotation. Differential expression analysis was performed using the Deseq2 package in R post-normalization based on a Benjamini-Hochberg false discovery rate (FDR)-corrected threshold for statistical significance of p value <0.01 and log_2_FC >1 The count of differentially expressed genes was used to generate heatmaps using Clustvis software. For gene set enrichment analysis, the 112 RelA target genes identified by Ngo et al (27) were used for GSEA analysis of the enrichment of the Deseq2 package normalized RNA-Seq data.

### High-Throughput Repertoire and SHM Analysis

To analyze the repertoire usage and SHM in the V_186.2_ region DNA, RNA was extracted from splenic B cells for cDNA synthesis, as described above. Rearranged V_186.2_DJ_H_-Cμ and V_186.2_DJ_H_-Cγ1 cDNA was amplified using Phusion™ high-fidelity DNA polymerase (New England BioLabs) and a V_186.2_ leader-specific forward primer and a reverse primer specific for the Cμ or Cγ1 exon (**Supplementary Table 2**), followed by the second round of PCR using the same forward primer and a nest reverse primer tagged with an Illumina clustering adapter (**Supplementary Table 2**), using the following protocol: 98°C for 10 s, 60°C for 45 s and 72°C for 1 m for 30 cycles. The amplified library was tagged with barcodes for sample multiplexing and analyzed by 300-bp pair ended sequencing (the Illumina Mi-Seq system).

The BCR repertoire usage and mutations in V_186.2_ (V1-72) segments were analyzed using the web-interfaced International ImmunoGeneTics Information System^®^ IMGT*/*HighV*-*QUEST (http://www.imgt.org). The mutation collection process is described in our previous study (28). Briefly, IgBLAST v1.15.0 (http://www.ncbi.nlm.nih.gov/igblast/) was applied for the alignment of the datasets of SHM sequencing (29). Change-O v1.0.0 (https://changeo.readthedocs.io/en/stable/) python package was applied for processing the output of V(D)J sequencing data (30). For mutation counting, Fasta.fmt7 files were generated by MakeDb.py with reference of imgt_mouse_ig_v.fasta, imgt_mouse_ig_d.fasta, imgt_mouse_ig_j.fasta and ParseDb.py was applied with --if SEQUENCE_ID --sf SEQUENCE_IMGT --mf V_CALL DUPCOUNT. Mutations in the V_186.2_ (IMGT V1-72) segment were filtered and aligned based on a single nucleotide with uncertain or missing bases replaced by "N" or "NA". The final step was applied by metric summary. Only unique sequences were further analyzed. The R pipeline was used to count point-mutations.

### Mitochondria-Free Cytosolic Fractionation, Immunoblotting, and Immunoprecipitation (IP) 

To prepare whole-cell lysates, B cells (10^7^) were harvested by centrifugation at 500 g for 5 m, resuspended in 0.5 ml of lysis buffer (20 mM Tris-Cl, pH 7.5, 150 mM NaCl, 0.5 mM EDTA, 1% (v/v) NP-40) supplemented with Halt™ Protease & Phosphatase Inhibitors Cocktail (ThermoScientific). To prepare mitochondria-free cytosolic fractions, B cells (10^7^) were centrifuged at 500 g for 5 m. Cell pellets were processed to obtain the mitochondria-free cytosolic fraction using the Mitochondrial Isolation Kit (ThermoFisher, Cat# 89874) following the manufacturer’s instructions. The whole cell lysates and the mitochondria-free cytosolic fraction were subjected to SDS–PAGE, and immunoblotting involving specific Abs (**Supplementary Table 1**). Membranes were then stripped with Restore™ PLUS Western Blot Stripping Buffer (ThermoScientific) for re-immunoblotting. Signals were quantified by ImageJ^®^ (NIH).

For IP, spleen B cells (10^7^) were resuspended in lysis buffer (20 mM Tris-Cl, pH 7.5, 100 mM NaCl, 1 mM EDTA, 0.5% (v/v) NP-40) supplemented with Halt™ Protease and Phosphatase Inhibitors Cocktail. After sonication and centrifugation, protein lysates were precleared with equilibrated Protein A/G-conjugated Sepharose™ 4B beads (ThermoFisher, 50 μl) and incubated with anti–cFLIP mouse Ab in 500 μl of lysis buffer at 4°C for 4 h in the presence of Protein A/G Sepharose™ 4B beads. After washing with lysis buffer 3 times, immunoprecipitated proteins were eluted in SDS sample buffer for immunoblotting.

### HOIPIN-8 Synthesis and Drug Treatment

HOIPIN-8 (sodium (E)-2-(3-(2,6-difluoro-4-(1H-pyrazol-4-yl)phenyl)-3-oxoprop-1-en-1-yl)-4-(1-methyl-1H-pyrazol-4-yl)benzoate) was synthesized using a two-step process. First, 1-(4-bromo-2,6-difluorophenyl)ethan-1-one (**Compound 1**; 1g, 4.26 mmol) was dissolved in the dioxane-H_2_O mixture (47 and 6 ml, respectively) in a 150-ml pressure vessel flask together with 4-(4,4,5,5-tetramethyl-1,3,2-dioxaborolan-2-yl)-1H-pyrazole (1g, 5.19 mmol), K_2_CO_3_ (1.76g, 12.77 mmol) and [1,1’-Bis(diphenylphosphino)ferrocene]dichloropalladium (II) complexed with dichloromethane (348 mg, 0.426 mmol). After the atmosphere in the flask was displaced with argon gas five times, the mixture was stirred and heated at 100°C for 16 h. The reaction was cooled to RT, quenched with HCl (12 ml, 2M), diluted with ethyl acetate (EtOAc), and filtered through celite. The organic layer of the filtrate was further separated and dried over Na_2_SO_4_. After filtration and concentration, the residue was purified by flash chromatography using Hexanes:EtOAc mixture (v/v=2/1 to 1/1), yielding 1-(2,6-difluoro-4-(1H-pyrazol-4-yl)phenyl)ethan-1-one (**Compound 2**; 945 mg, quantitative yield) with the following profile: ^1^H NMR (400 MHz, cdcl_3_) δ 7.88 (s, 2H), 7.06 (d, *J* = 9.5 Hz, 2H), 2.55 (s, 3H); ESI-MS: m/z 223.2 [M+H]+.

In a separate reaction, 3-Hydroxy-5-(1-methyl-1H-pyrazol-4-yl)isobenzofuran-1(3H)-one (**Compound 5**) was synthesized, starting from 5-bromoisobenzofuran-1(3H)-one (**Compound 3**) and involving the intermediate 5-bromo-3-hydroxyisobenzofuran-1(3H)-one (**Compound 4**), following a published protocol (25).

**Table:** 

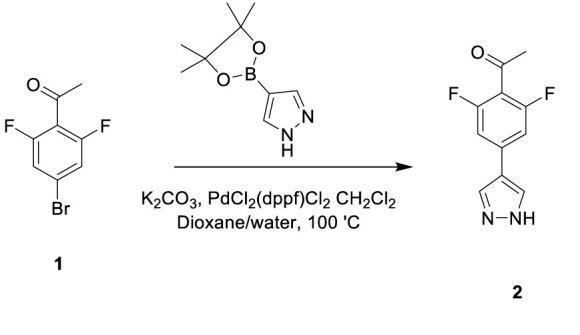

**Table:** 

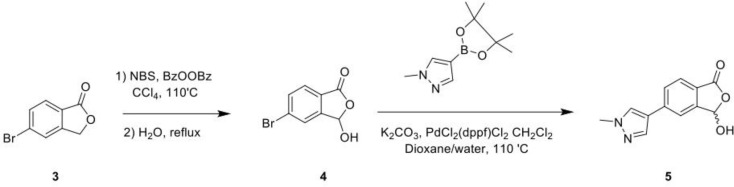

In the second step, Compound 2 (483 mg, 2.17 mmol) and Compound 5 (500 mg, 2.17 mmol) were dissolved in EtOH (12 mL) in a 100-ml glass round-shaped flask. The suspension was cooled to 0°C and, with NaOH (2.9 ml, 6M, 17.34 mmol in total) added, allowed to warm to RT. The mixture was stirred for 16 h until the reaction was quenched with HCl (12 ml, 2M), transferred to a separator funnel, and extracted with chloroform:isopropanol (3:1) three times. The organic layers were collected and dried over Na_2_SO_4._ After filtration and concentration, the residue was purified by C18 reversed phase flash chromatography using a 0-55% acetonitrile:water gradient to give the HOIPIN-8 base. Finally, the (HOIPIN-8) freebase (80 mg, 0.184 mmol) was dissolved in EtOH (1.5 ml) in a 100-ml glass round-shaped flask, added NaOH (0.2 mL, 1M, 0.184 mmol) at 0°C and stirred for 1 h, warmed to RT and stirred for 2 h. The suspension was then lyophilized to obtain HOIPIN-8 a yellow powder (25% yield) with the following profile: ^1^H NMR (400 MHz, dmso) δ 8.78 (d, *J* = 16.3 Hz, 1H), 8.32 – 8.19 (m, *J* = 8.9 Hz, 3H), 7.92 (d, *J* = 21.3 Hz, 2H), 7.57 (d, *J* = 8.0 Hz, 1H), 7.54 – 7.47 (m, 3H), 7.13 (d, *J* = 16.2 Hz, 1H), 3.86 (s, 3H); ESI-MS: m/z 435.4 [M+H]+.

**Table:** 

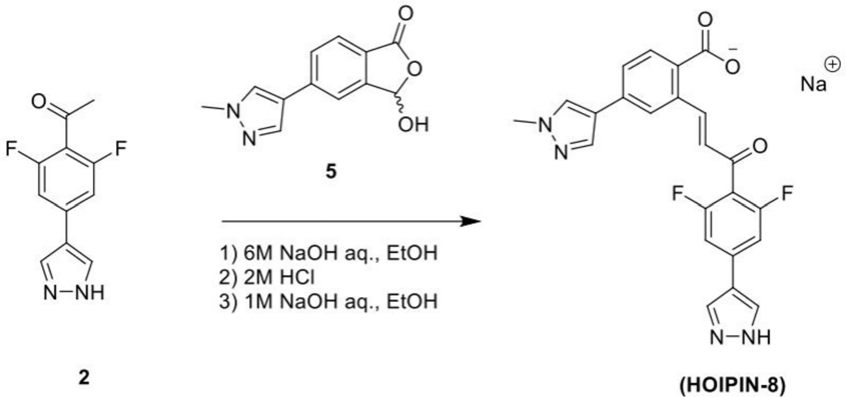

To treat B cells with HOIPIN-8, lyophilized HOIPIN-8 was dissolved in DMSO to yield a stock solution of 40 mM, which was further diluted in RPMI-FBS and added to the cell culture medium, with a final concentration of 20 μM or as indicated. To treat B cells with Z-VAD-FMK, Z-LEHD-FMK, or Z-IETD-FMK, these compounds, as dissolved in DMSO, were added to B cell cultures at indicated concentrations.

### Immunofluorescence Imaging

Spleens were embedded in OCT (Tissue-Tek) and snap-frozen on dry ice. Cryostat sections (5 μm) were fixed in pre-chilled acetone for 10 m, air dried at 25°C, washed with PBS, and blocked with 5% FBS in DPBS for 1 h. Sections were stained with FITC-conjugated anti-B220 mAb (1:500) and PerCP-Cy5.5-conjugated anti-GL-7 mAb (1:100) in a humidified chamber overnight at 4°C. Slides were mounted using ProLong® Gold with DAPI for analysis under a Zeiss LM710 confocal microscope. All images are pseudocolored.

### Statistical Analysis

Statistical analysis was performed by either GraphPad (Prism®) or Excel (Microsoft) software to determine *p* values by Student t-test. *p* values less than 0.05 were considered significant. Correlation analyses were performed using Prism®.

## Results

### B-Cell SHARPIN Promotes T-Dependent Ab Responses and a BCR Affinity-Enhancing Mutation

**Figure 1:**
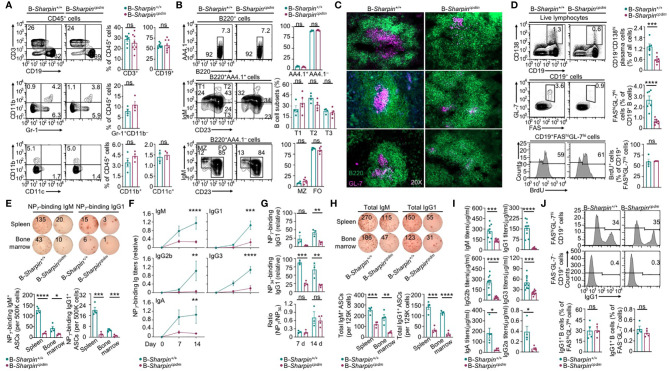
B cell-intrinsic role of SHARPIN in the GC development and Ab responses. **(A, B)** Flow cytometry analysis of different immune cell populations, as indicated, in the spleen of B-*Sharpin^+/+^* or B-*Sharpin^cpdm^*mice immunized with NP-CGG/alum for 14 d. **(C)** Fluorescence imaging analysis of GCs in the spleen in mice, as in **(A, B)**. **(D)** Flow cytometry analysis of plasma cells, GC B cells, and GC B cell proliferation (by BrdU incorporation) in immunized mice. **(E–G)** ELISPOT analyses of ASCs producing NP-specific IgM or IgG1 in the spleen and the bone marrow in mice 14 d after immunization **(E)** and ELISA of circulating NP_7_-binding and NP_34_-binding Ig Abs 7 and 14 d after immunization **(F, G)**. **(H, I)** ELISPOT analyses of ASCs producing total IgM or IgG **(H)** and ELISA of circulating total Ig Abs **(I)** in mice, as in **(E, F)**. Data are pooled from 2 independent experiments. **(J)** Flow cytometry analysis of surface IgG1 expression in GC and non-GC B cells. *p<0.05; **p<0.01; ***p<0.001; ****p<0.0001; ns, not significant; t-test.

Like B cell-specific ablation of HOIP in *mb1^+/cre^Hoip^fl/fl^* mice (31), B cell-specific deficiency in SHAPRIN in B-*Sharpin^cpdm^* mice did not affect the maturation of follicular or marginal zone B cells or other immune cells, including T cells, dendritic cells (DCs), macrophages and neutrophils (**Supplementary Figure 1A** and **Figures 1A, B**). Upon injection with alum-mixed NP-CGG, which elicits a T-dependent Ab response, B-*Sharpin^cpdm^* mice showed severe impairment in the GC development in the spleen and the output of FAS^hi^GL-7^hi^ GC B cells and CD138^+^ plasma cells despite normal GC and total B cell proliferation (**Figures 1C, D** and **Supplementary Figure 1B**), culminating in decreased formation of ASCs that produced NP-binding IgM and IgG1 Abs as well as much reduced titers of NP-specific IgM and high-affinity (NP_7_-binding) IgG1, IgG2b, IgG3 and IgA (**Figures 1E, F**) – total NP-specific (NP_34_-binding) IgG1 titers were also reduced to similar extents (**Figure 1G**). ASCs secreting non-specific IgM and IgG1 were reduced too, resulting in decreased titers of circulating IgM and IgG1 – also decreased were IgG2a and IgA, as elicited by both T-dependent and T-independent antigens, as well as IgG3 and IgG2b, as elicited mainly by T-independent antigens (**Figures 1H, I**). Residual B-*Sharpin^cpdm^* GC B cells, however, expressed IgG1 at high levels, indicating their normal CSR (**Figure 1J**).

**Figure 2:**
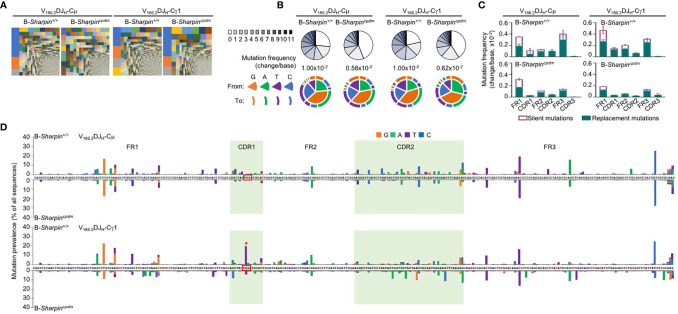
SHARPIN mediates the generation of high-affinity BCR mutants. **(A)** Treemap charts depicting B cell clones identified by unique CDR3 sequences in the V_H_ region of V_186.2_DJ_H_-Cμ and V_186.2_DJ_H_-Cγ1 in B-*Sharpin^+/+^* and B-*Sharpin^cpdm^*mice 14 d after NP-CGG/alum immunization. Each rectangle represents a unique clone and the size of each rectangle depicts the relative abundance of the clone within the total population. **(B)** Proportions of V_186.2_DJ_H_-Cμ and V_186.2_DJ_H_-Cγ1 clones carrying given numbers of point-mutations in V_186.2_ region in mice, as in **(A)**, as depicted by pie chart slices (top row; sequences with over 12 point-mutations were excluded from analysis), the overall mutation frequencies (middle), and the nature of such point-mutations, as depicted by concentric pie chart slices and rings (bottom row). **(C,D)** Histograms depict the silent and replacement mutations in different CDRs and FRs as well as in the entire V_186.2_DJ_H_-Cμ and V_186.2_DJ_H_-Cγ1 regions **(C)** and the spectrum and distribution of point-mutations (**D**, “*” denotes the G98->T98 mutation) in mice, as in **(A,B)**. Data are pooled from three mice in each group.

Upon NP-CGG immunization, B cells with a recombined IgH V_186.2_ region, which encodes NP-binding BCRs, enter GCs and accumulate V_186.2_ DNA mutations to generate BCR mutants for selection (32–34). As shown by analysis of IgM-encoding V_186.2_DJ_H_-Cμ and IgG1-encoding V_186.2_DJ_H_-Cγ1 transcript, NP-CGG-immunized B-*Sharpin^cpdm^* mice had similar BCR repertoires in IgG1^+^ B cells as B-*Sharpin^+/+^* mice, but more diverse ones in IgM^+^ B cells (**Figure 2A** and **Supplementary Figure 1C**). V_186.2_ DNA mutations occurred at high levels in V_186.2_DJ_H_-Cγ1 of both B-*Sharpin^+/+^* and B-*Sharpin^cpdm^* mice, averaging 1.0x10^–2^ and 0.6x10^–2^ change per base, respectively (**Figure 2B** and **Supplementary Figure 1D**). They also frequently occurred in V_186.2_ DJ_H_-Cμ and featured G→A and C→T transitions as major substitutions (**Figure 2B**), likely due to the “replication over” of uracils generated after AID deamination of cytidines (pairing “A” with “U” instead of “G” with the original C). Such transitions were also dominant, albeit not as much, in V_186.2_DJ_H_-Cγ1, likely reflecting mutation spreading by error-prone DNA synthesis.

In both B-*Sharpin^+/+^* and B-*Sharpin^cpdm^* mice, base changes in CDR1 and CDR2 of V_186.2_ DJ_H_-Cγ1 DNA were dominated by replacement mutations (**Figure 2C**), consistent with the notion that amino acid residues in CDRs are altered to serve as the substrates for selection. In particular, a G→T transversion substitution (G_98_→T_98_) changes the CDR1 Trp_33_ residue (TGG) to Leu (TTG) to raise the BCR/Ab affinity to NP by 10-fold (34, 35). It was the most frequent mutation within the CDR regions of V_186.2_ DJ_H_-Cγ1 in B-*Sharpin^+/+^* mice, occurring in 19.7% of B cells in these mice, much higher than the 4.18% frequency in B-*Sharpin^cpdm^* B cells – all other CDR mutations showed smaller differences (**Figure 2D** and **Table 1**). Furthermore, among the ten most frequent mutations in the entire V_186.2_ DJ_H_-Cγ1 DNA, all but G_98_→T_98_ were in the FR regions (five in FR1, one in FR2, and three in FR3) and showed less difference between B-*Sharpin^+/+^* and B-*Sharpin^cpdm^* mice (**Figure 2D** and **Table 1**). In addition, G_98_→T_98_ was unique to V_186.2_ DJ_H_-Cγ1 DNA, in contrast to the frequent occurrence of all other dominant mutations also in V_186.2_ DJ_H_-Cμ DNA (**Figure 2D**). Finally, while the CDR1 in V_186.2_ DJ_H_-Cμ did not show overall enrichment of replacement mutations (**Figure 2C**), the CDR1 G_92_→A_92_ and CDR2 G_197_→A_197_ or T_197_ replacement mutations occurred much less frequently in B-*Sharpin^cpdm^* mice (**Figure 2D** and **Table 1**).

**Table 1:** Distribution and frequencies of dominant V_186.2_ mutations in B-*Sharpin^+/+^* and B-*Sharpin^cpdm^* mice immunized with NP-CGG and the ratio of mutation frequencies in B-*Sharpin^+/+^* mice to those in B- *Sharpin^cpdm^* mice.

V_186.2_DJ_H_-Cμ, CDRs				V_186.2_DJ_H_-Cμ, FRs			
Mutation	Frequency (x10^–2^ change/base)		Ratio	Mutation	Frequency (x10^–2^ change/base)		Ratio
/region	B-*Sharpin^+/+^*	B-*Sharpin^cpdm^*		/region	B-*Sharpin^+/+^*	B-*Sharpin^cpdm^*	
AG_92_C/CDR1^†^	4.04	0.29	14.1	CTT_33_/FR1	16.8	17.4	0.97
AGC_93_/CDR1^†*^	6.00	1.89	3.17	GTG_36_/FR1	6.55	5.86	1.12
CAC_105_/CDR1	3.39	3.73	0.91	AA_38_G/FR1	10.6	13.0	0.82
AG_149_G/CDR2	5.24	5.03	1.04	G_46_CT/FR1	6.41	6.76	0.95
G_154_AT/CDR2	4.85	4.57	1.06	C_58_TG/FR1	9.87	8.53	1.16
A_160_AT/CDR2	6.67	0.90	7.45	CG_128_A/FR2	8.72	8.72	1.00
G_169_GT/CDR2	5.94	5.24	1.13	C_208_TG/FR3	7.00	8.41	0.83
AAG_177_/CDR2	8.70	7.70	1.13	C_223_CC/FR3	19.3	19.5	0.99
A_196_GC/CDR2	5.36	6.70	0.80	CAG_246_/FR3	15.8	7.32	2.16
AG_197_C/CDR2	12.7	2.26	5.62	TAT_285_/FR3	25.4	27.8	0.91
**V_186.2_DJ_H_-Cγ1, CDRs^#^**				**V_186.2_DJ_H_-Cγ1, FRs^#^**			
**Mutation**	**Frequency (x10^–2^ change/base)**		**Ratio**	**Mutation**	**Frequency (x10^–2^ change/base)**		**Ratio**
**/region**	**B-*Sharpin^+/+^***	**B-*Sharpin^cpdm^***		**/region**	**B-*Sharpin^+/+^***	**B-*Sharpin^cpdm^***	
AG_92_C/CDR1^†^	4.95	7.93	0.62	GAG_30_/FR1^*^	11.7	5.36	2.18
TG_98_G/CDR1^‡^	19.7	4.18	4.71	CTT_33_/FR1^*^	22.6	8.75	2.58
CAC_105_/CDR1	6.96	2.10	3.31	AA_38_G/FR1	17.8	6.96	2.56
AG_149_G/CDR2	3.34	2.46	1.36	G_46_CT/FR1	11.6	4.60	2.52
G_154_AT/CDR2	2.34	1.79	1.31	C_58_TG/FR1	10.4	5.16	2.02
AAG_177_/CDR2	4.96	2.81	1.77	AAG_114_/FR2	4.43	2.06	2.15
G_184_AG/CDR2	3.75	1.77	2.12	CG_128_A/FR2	10.4	5.43	1.92
GAG_186/_CDR2	3.90	5.11	0.76	C_208_TG/FR3^*^	9.79	10.6	0.92
A_196_GC/CDR2	7.43	2.96	2.51	C_223_CC/FR3	16.6	16.3	1.02
AG_197_C/CDR2	7.87	12.9	0.61	TAT_285_/FR3^*^	24.8	8.11	3.06

^†^The AG92C93 (Ser) codon has a high mutability and all mutations are replacement mutations except for AGT93 (36).

^‡^The TG98G (Trp) to TT98G (Leu) replacement mutation changes the affinity of Abs to NP by 10-fold (34,35). The frequency of G98 ®T98 mutation were 33.6x10–2, 11.0 x10–2, and 9.8x10–2 change/base in three B-Sharpin+/+ mice, and 7.8 x10–2, 2.9x10–2, and 0.2x10–2 change/base in three B-Sharpincpdm mice. Since only unique sequences were analyzed, the frequencies of each mutation were equivalents of the proportions of B cell clones carrying such mutations.

^#^The mutations depicted here had the highest frequencies in the CDRs and FRs, respectively, in B-Sharpin+/+ mice. Additional dominant mutations occurred at high frequencies in B-Sharpincpdm mice (6 in FR1, 4 in CDR2, and 3 in FR3).

*The mutations are all silent mutations.

Thus, SHARPIN plays a B cell-autonomous role in promoting the GC reaction, accumulation of the affinity-augmenting G_98_→T_98_ substitution and maturation of the T-dependent Ab response.

### B Cell-Intrinsic Role of SHARPIN in Promoting GC B Cell Survival

**Figure 3:**
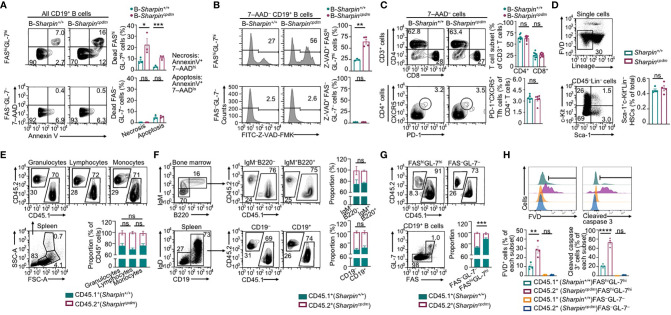
SHARPIN is critical for germinal center B cell survival. **(A,B)** Flow cytometry analysis of apoptosis and necrosis **(A)** as well as caspase activation **(B)** in FAS^hi^GL-7^hi^ GC B cells and FAS^–^GL-7^–^ non-GC B cells in the spleen of B-*Sharpin^+/+^* or B-*Sharpin^cpdm^*mice 14 d after immunization with NP-CGG/alum. **(C)** Flow cytometry analysis of CD4^+^ and CD8^+^ T cells (top panels) as well as CD4^+^CXCR5^+^PD-1^+^ Tfh cells (bottom) in mice, as in **(A,B)**. **(D)** Gating strategy for flow cytometry analysis of Sca-1^+^c-Kit^+^Lin^–^ HSCs and the proportion of such cells in the bone marrow of *Sharpin^+/+^* and *Sharpin^cpdm^* mice. **(E)** Flow cytometry analysis of (SSC^hi^FSC^hi^) granulocytes, (SSC^lo^FSC^lo^) lymphocytes, and (SSC^lo^FSC^hi^) monocytes in the spleen of CD45.1^+^ *Sharpin^+/+^/*CD45.2^+^ *Sharpin^cpdm^* mixed bone marrow chimera mice (mean and s.d., n=4). **(F)** Flow cytometry analysis of the proportions of CD45.1^+^ and CD45.2^+^ cells in different B cell subsets in the bone marrow and spleen of CD45.1^+^ *Sharpin^+/+^/*CD45.2^+^ *Sharpin^cpdm^* mice (mean and s.d., n=4). **(G, H)** Flow cytometry analysis of proportions of CD45.1^+^ and CD45.2^+^ cells in FAS^hi^GL-7^hi^ GC B cells and FAS^–^GL-7^–^ non-GC B cells **(G)** as well as survival and caspase 3 cleavage **(H)** in such cells in CD45.1^+^ *Sharpin^+/+^/*CD45.2^+^ *Sharpin^cpdm^* mice 14 d after NP-CGG/alum immunization (mean and s.d., n=4). * *p*<0.05; ** *p*<0.01; *** *p*<0.001; **** *p*<0.0001; t-test.

B cell clones with higher-affinity BCRs need to survive in GCs towards maturation of the Ab responses. In association with much-decreased high-affinity NP-specific IgG Abs and enrichment of the affinity-enhancing G_98_→T_98_ mutation in B-*Sharpin^cpdm^* mice, FAS^hi^GL-7^hi^ GC B cells in these mice had increased death, including more apoptosis and necrosis (**Figure 3A**). Among live B-*Sharpin^cpdm^* GC B cells, more than half showed caspase activation (Z-VAD-FMK^+^), a hallmark of pre-apoptotic and apoptotic cells (**Figure 3B**). The proportions of apoptotic, necrotic, and Z-VAD-FMK^+^ cells in non-GC (FAS^–^GL-7^–^) B cells were all much lower than those in GC B cells in both B-*Sharpin^+/+^* and B-*Sharpin^cpdm^* mice. They were comparable in these mice, showing that SHARPIN deficiency selectively affects GC B cell survival.

Tfh cells, which are thought to mediate the GC B cell survival (37), were normal in B-*Sharpin^cpdm^*mice (**Figure 3C**), suggesting that B-*Sharpin^cpdm^* GC B cells had intrinsic defects to survive in an otherwise normal microenvironment. This was addressed in *Cd45.1^+^-Sharpin^+/+^*/*Cd45.2^+^-Sharpin^cpdm^* chimera mice, as generated by γ-irradiated C57 mice engrafted with an equal number of *Cd45.1^+^-Sharpin^+/+^* and *Cd45.2^+^-Sharpin^cpdm^* bone marrow cells, which had comparable numbers of hematopoietic stem cells (HSCs; **Figure 3D**). In such mice, *Cd45.2^+^-Sharpin^cpdm^* HSCs gave rise to 25-30%, instead of the expected 50% of cells in different leukocyte compartments, including IgM^+^B220^+^ immature B cells and IgD^+^ mature B cells (**Figures 3E, F**), likely due to a partial defect of *Sharpin^cpdm^* HSCs in differentiation. Upon NP-CGG immunization, the chimera mice supported the development of GC B cells, of which only 10% were *Cd45.2^+^-Sharpin^cpdm^*, significantly less than the 30% of *Cd45.2^+^-Sharpin^cpdm^* B cells within the non-GC B cell compartment (**Figure 3G**). In association with the underrepresentation of *Cd45.2^+^-Sharpin^cpdm^* GC B cells, 28% of such cells lost membrane integrity (FVD^+^) and, among FVD^–^ cells, 70% showed caspase 3 cleavage, both reflecting over 2-fold increases in corresponding cell frequencies in *Cd45.1^+^-Sharpin^+/+^* GC B cell counterparts (**Figure 3H**). Such increases were also in line with the fold of increase in Annexin V^+^7–AAD^lo^ apoptotic and Z-VAD-FMK^+^ pre-apoptotic/apoptotic GC B cells in B-*Sharpin^cpdm^*mice (**Figures 3A, B**). By contrast, non-GC *Cd45.2^+^-Sharpin^cpdm^* B cells were virtually all FVD^–^ and showed no caspase 3 activation, confirming that the defect of *Sharpin^cpdm^* B cells in survival was specific to GC B cells.

Overall, SHARPIN plays a B cell-intrinsic role in inhibiting apoptosis of GC B cells and mediating their survival during the T-dependent Ab response.

### SHARPIN Suppresses IL-21-Induced Death in CD154-Stimulated B Cells

**Figure 4:**
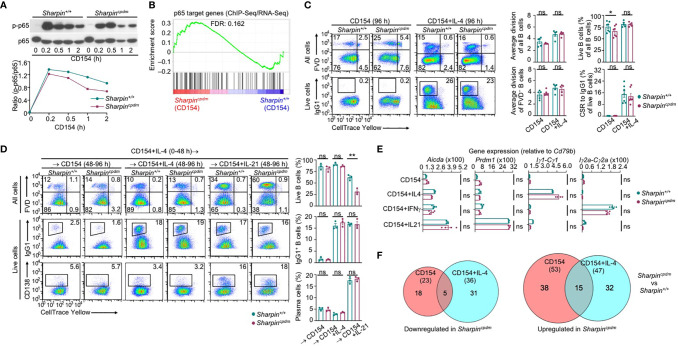
SHARPIN is dispensable for activation, proliferation, survival, and differentiation of B cells stimulated with CD154. **(A)** Immunoblotting of phosphorylated p65 and total p65 protein levels in *Sharpin^+/+^* and *Sharpin^cpdm^* B cells after stimulation by CD154 for the indicated time. Representative of two independent experiments. **(B)** GSEA analysis of differentially expressed genes (DEGs) in *Sharpin^+/+^* and *Sharpin^cpdm^* B cells stimulated with CD154 for 24 h for the enrichment of p65 target genes (RNA-Seq data were pooled from two independent experiments). **(C)** Flow cytometry analysis of proliferation, survival, and CSR to IgG1 in *Sharpin^+/+^* and *Sharpin^cpdm^* B cells stimulated with CD154 or CD154 plus IL-4 for 96 h. **(D)** Flow cytometry analysis of survival, CSR to IgG1 and plasma cell differentiation in *Sharpin^+/+^* and *Sharpin^cpdm^* B cells after stimulation first with CD154 plus IL-4 for 48 h, washed, and then with CD154, alone or plus IL-4 or IL-21, for 48 h. **(E)** qRT-PCR analysis of gene expression, as indicated, in B cells stimulated with CD154, alone or plus IL-4, IFN-γ or IL-21, for 48 h. **(F)** The numbers of DEGs (*p* values < 0.01, absolute value of log_2_FC > 1) between *Sharpin^+/+^* and *Sharpin^cpdm^* B cells after stimulation with CD154 or CD154 plus IL-4 for 24 h, as depicted by Venn diagram. Data were pooled from two independent RNA-Seq experiments. * *p*<0.05; ** *p*<0.01; t-test.

To understand the mechanisms underlying the increased death of *Sharpin^cpdm^* GC B cells, we subjected purified *Sharpin^cpdm^*B cells to stimulation by membrane-bound CD154, which mimics CD154 expressed on the plasma membrane of Tfh cells that potently engages CD40 to initiate and sustain the GC reaction (6, 38, 39). CD154-stimulated *Sharpin^cpdm^*B cells only mildly impaired in canonical NF-κB activation, as indicated by phosphorylation of the p65 subunit at Ser333, and were largely normal in expressing p65 target genes (**Figures 4A, B**). Consequently, they showed normal proliferation and IL-4-directed CSR to IgG1 (**Figure 4C**). While neither *Sharpin^+/+^* nor *Sharpin^cpdm^* B cells differentiated into plasma cells at a high level after 96 h of stimulation with CD154 and IL-4, they both could do so after 48 h of stimulation, and being washed to remove IL-4 and re-stimulation for 48 h with CD154 and IL-21 (**Figure 4D**). Accordingly, CD154-stimulated *Sharpin^cpdm^* B cells were fully capable of inducing *Prdm1* (encoding BLIMP-1) and upregulating AID in the presence of IL-21 as well as undergoing IL-4- and IFNγ-directed Iγ1-Cγ1 and Iγ2a-Cγ2a germline transcription, respectively (**Figure 4E**). They also shared a similar transcriptome with their *Sharpin^+/+^* B cell counterparts, with only 76 (23 downregulated and 53 upregulated) and 83 (36 downregulated and 47 upregulated) differentially expressed genes upon stimulation by CD154 and CD154 plus IL-4, respectively (**Figure 4F** and **Supplementary Figure 2A**). Finally, *Sharpin^cpdm^* B cells underwent robust proliferation, CSR, and plasma cell differentiation upon stimulation by LPS (**Supplementary Figure 2B**), showing that SHARPIN did not mediate B cell responses to T-dependent or T-independent stimuli.

**Figure 5:**
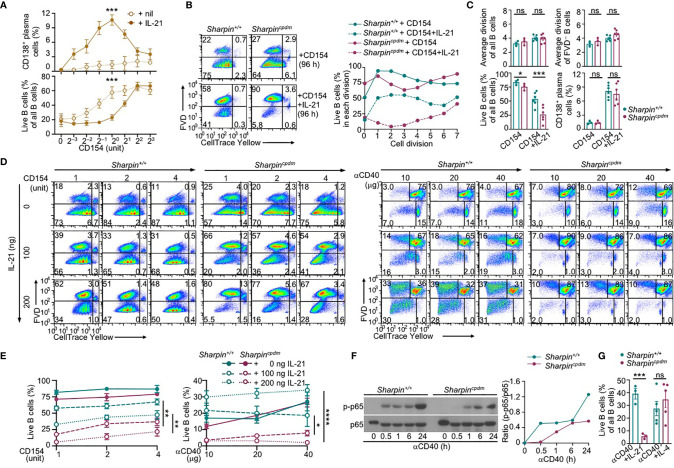
SHARPIN deficiency exacerbates IL-21-induced death in CD154-stimulated B cells. **(A)** Flow cytometry analysis of plasma cell differentiation and B cells viability in C57 B cells stimulated with different doses of CD154, as indicated, in the absence or presence of IL-21 for 96 h (n=4, mean and s.e.m.; data at 1 unit of CD154 were analyzed for statistical differences between *Sharpin^+/+^* and *Sharpin^cpdm^* B cells). **(B, C)** Flow cytometry analysis of proliferation, survival, and plasma cell differentiation of *Sharpin^+/+^* and *Sharpin^cpdm^* B cells stimulated with CD154 or CD154 plus IL-21 for 96 (h) Division-linked B cell viability is depicted in **(B)**, representative of four independent experiments) and average divisions, live B cell proportions, and CD138^+^ plasma cell proportions were depicted in **(C)**. **(D, E)** Flow cytometry analysis of proliferation, survival of *Sharpin^+/+^* and *Sharpin^cpdm^* B cells stimulated with CD154 (left panels) or αCD40 (right panels) at indicated doses in the presence of different doses of IL-21 for 96 h (n=3, mean and s.e.m.; data at 4 units of CD154 or 40 μg of αCD40 were analyzed for statistical differences between *Sharpin^+/+^* and *Sharpin^cpdm^* B cells). **(F)** Immunoblotting of phosphorylated p65 and total p65 protein levels in *Sharpin^+/+^* and *Sharpin^cpdm^* B cells after stimulation by αCD40 for the indicated time. Representative of two independent experiments. **(G)** Live cell proportions in *Sharpin^+/+^* and *Sharpin^cpdm^* B cells stimulated with αCD40 plus IL-21 or IL-4 for 96 (h) *p < 0.05; **p < 0.01; ***p < 0.001; ****p < 0.0001; t-test.

IL-21 enhanced proliferation and induced plasma cell differentiation of CD154-stimulated B cells, but triggered their death, resulting in a net loss of such cells (**Figures 5A–C**). The killing effect of IL-21 was exacerbated by the *Sharpin* deficiency, leading to as few as 10% of *Sharpin^cpdm^*B cells able to survive, virtually all of which had completed high numbers of division (**Figures 5B, C**). By contrast, *Sharpin^cpdm^* B cells stimulated with CD154 alone or with IL-4 showed high viability, which was only marginally lower than that of *Sharpin^+/+^* B cells (**Figure 4C**). *Sharpin^cpdm^* B cells were also normal in survival upon stimulation by αIgM plus IL-4, but defective upon stimulation IL-21 alone, LPS or LPS plus IL-4 – αIgM (alone or with IFNγ) or LPS plus IL-21 could not maintain the viability of even wildtype B cells (**Supplementary Figure 2B–D**). High doses of CD154 countered the death-inducing effect of IL-21 in *Sharpin^+/+^* B cells and more effectively in *Sharpin^cpdm^* B cells (**Figures 5A, D, E**), showing a SHARPIN-independent pro-survival activity of strong CD40 signals.

CD40 could be engaged *in vitro* by αCD40, which activated NF-κB in wildtype B cells, albeit not as quickly or potently as CD154 (**Figures 4A**, **5F**), and require IL-21 to induce these B cells to proliferate (**Figures 5D, E**). More than half of dividing *Sharpin^+/+^* cells, however, were unable to survive, even in the presence of high doses of αCD40. αCD40-stimulated *Sharpin^cpdm^* B cells showed much weaker NF-κB activation at early time points when B cells were particularly sensitive to the killing by IL-21 (**Figure 5F**) and failed to robustly proliferate even in the presence of IL-21, which instead triggered death in both dividing and non-dividing B cells (**Figures 5D, E**). Upon stimulation with αCD40 plus IL-4, *Sharpin^cpdm^*, by contrast, showed comparable survival as *Sharpin^+/+^* B cells (**Figure 5G**).

To summarize, SHARPIN does not play a major role in CD154-induced B-cell NF-κB activation, gene expression, proliferation, CSR, or plasma cell differentiation. Rather, it specifically suppresses IL-21-induced B cell death through a mechanism not entirely overlapping with the one underlying the effect of CD154 in countering the killing by IL-21.

### SHARPIN Inhibits Caspase 8 and Caspase 9 Activation and B Cell Apoptosis 

**Figure 6:**
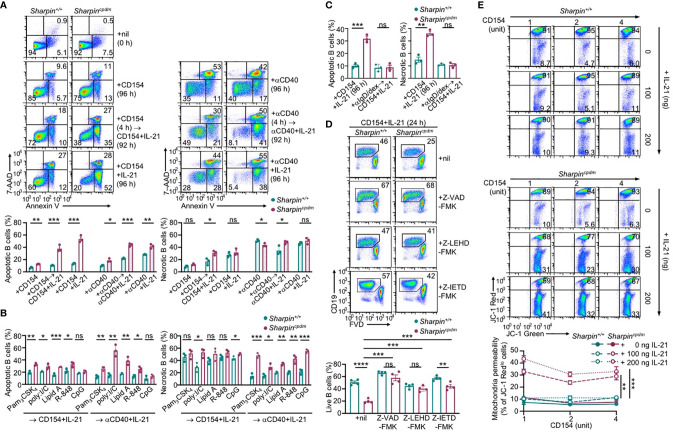
SHARPIN inhibits IL-21-induced apoptosis in CD154-stimulated B cells. **(A)** Flow cytometry analysis of apoptosis (Annexin V^+^7–AAD^lo^) and necrosis (Annexin V^+^7–AAD^hi^) in freshly isolated *Sharpin^+/+^* and *Sharpin^cpdm^* B cells or after stimulation with CD154 (left) or αCD40 (right) plus nil or IL-21 for 96 h, with or without priming with CD154 or αCD40 for 4 h. **(B, C)** Flow cytometry analysis of apoptosis and necrosis in *Sharpin^+/+^* and *Sharpin^cpdm^* B cells after priming with TLR ligands (as indicated, **B**) or αIgD/dex **(C)** for 4 h and then stimulated with CD154 or αCD40 plus IL-21 for 92 h. **(D)** Flow cytometry analysis of the viability of *Sharpin^+/+^* and *Sharpin^cpdm^* B cells stimulated with CD154 plus IL-21 in the presence of nil or pan-caspase inhibitor Z-VAD-FMK, caspase 9-specific inhibitor Z-LEHD-FMK or caspase 8-specific inhibitor Z-IETD-FMK (all 20 μM). **(E)** Mitochondrial membrane potential in B cells stimulated with CD154 and IL-21 at indicated doses for 48 h (n=4, mean and s.e.m.; data at 4 units of CD154 were analyzed for statistical differences between *Sharpin^+/+^* and *Sharpin^cpdm^* B cells). *p < 0.05; **p<0.01; ***p < 0.001; t-test.

IL-21 induced more apoptosis (AnnexinV^+^7–AAD^lo^) in CD154 or αCD40-stimulated *Sharpin^cpdm^* B cells than *Sharpin^+/+^*B cells, in addition to extensive but similar degrees of necrosis (AnnexinV^+^7–AAD^hi^) in both – αCD40 alone triggered more necrosis than CD154 (**Figure 6A**). The heightened apoptosis in *Sharpin^cpdm^* B cells could be alleviated by pre-treatment with CD154, BCR-crosslinking αIgD/dex or TLR9 ligand CpG, but not other TLR ligands, which instead reduced αCD40-triggered necrosis (**Figures 6A–C** and **Supplementary Figures 3A, B**). The viability of CD154 and IL-21-stimulated *Sharpin^cpdm^* B cells could be rescued by the pan-caspase inhibitor Z-VAD-FMK in a dose-dependent manner to a level comparable to that of *Sharpin^+/+^*B cells (**Figure 6D** and **Supplementary Figure 3C**). It could also be partially restored by Z-IETD-FMK and Z-LEHD-FMK, which specifically inhibits initiator caspase 8 and caspase 9, respectively (**Figure 6D** and **Supplementary Figure 3C**), showing that *Sharpin^cpdm^* B cell apoptosis was mediated by both the mitochondria-independent and mitochondria-dependent (intrinsic) apoptosis pathways. The integrity of mitochondria membranes was severely compromised by IL-21 in CD154-stimulated *Sharpin^cpdm^* B cells, but much less so in *Sharpin^+/+^*B cells (**Figure 6E**). This, together with the failure of high doses of CD154 to readily improve the mitochondria membrane integrity in either cell types, indicated that IL-21 overrode CD154 to activate the intrinsic apoptosis pathway that, if not controlled by SHARPIN, led to cell death.

**Figure 7:**
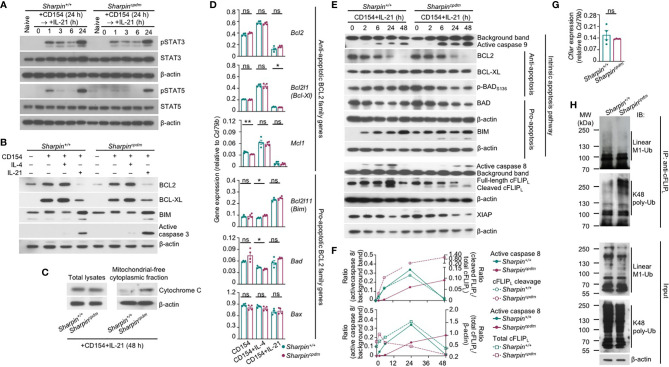
SHARPIN inhibits caspase 8 and caspase 9 activation in B cells stimulated with CD154 and IL-21. **(A)** Immunoblotting analysis of STAT3 and STAT5 activation in *Sharpin^+/+^* and *Sharpin^cpdm^* B cells stimulated with CD154 for 24 h and then IL-21 for different periods of time, as indicated. **(B, C)** Immunoblotting analysis of BCL2, BCL-XL and BIM expression and caspase 3 activation **(B)** and cytochrome C released into the cytosol **(C)** in *Sharpin^+/+^* and *Sharpin^cpdm^* B cells stimulated for 48 h. **(D)** qRT-PCR analysis of gene expression, as indicated, in *Sharpin^+/+^* and *Sharpin^cpdm^* B cells stimulated with CD154, alone or with IL-4 or IL-21, for 24 h (*p<0.05; **p < 0.01; t-test). **(E–F)** Immunoblotting analysis of caspase 8 and caspase 9 activation, expression of cFLIP_L_, XIAP and BCL2 family members as well as BAD activation in *Sharpin^+/+^* and *Sharpin^cpdm^* B cells stimulated with CD154 and IL-21 for different periods of time, as indicated. Quantified signals of caspase 8 activation, cFLIP_L_ cleavage and total cFLIP_L_ levels were depicted in **(F)**. **(G)** qRT-PCR analysis of *Cflar* expression in *Sharpin^+/+^* and *Sharpin^cpdm^* B cells stimulated with CD154 plus IL-21 for 24 h (t-test). **(H)** Proteins in whole cell lysates (input) and immunoprecipitated by anti-cFLIP were analyzed by immunoblotting for linear M1-Ub and K48 poly-Ub levels in *Sharpin^+/+^* and *Sharpin^cpdm^* B cells stimulated with CD154 plus IL-21 for 48 h. All immunoblotting analyses were representative of two independent experiments.

Like *Sharpin^+/+^*B cells, *Sharpin^cpdm^* B cells responded to IL-21 to activate STAT3 and STAT5, downregulate the expression of BCL2 and BCL-XL to supersede the effect of CD154 in inducing these anti-apoptosis members of the BCL2 family, and upregulate BIM, a pro-apoptotic member (**Figures 7A, B**). As compared to *Sharpin^+/+^*B cells, they also expressed comparable transcript levels of anti-apoptotic (*Bcl2, Bcl2l1/Bcl2-Xl*, *Mcl2*, which were all downregulated by IL-21) and pro-apoptotic (*Bcl2l11/Bim*, *Bad* and *Bax*) genes, all of which are involved in the regulation of mitochondrial membrane permeability, but showed more cytochrome C accumulation in the cytoplasm and activation of the effector caspase 3 (**Figures 7B–D**), indicating that SHARPIN deficiency amplified IL-21-triggered intrinsic apoptosis without altering the expression of the BCL2 family factors, likely by boosting downstream caspase activation. Indeed, upon IL-21 induction, CD154-stimulated *Sharpin^+/+,^*and *Sharpin^cpdm^* B cells underwent similar changes in the levels of BCL2, BCL-XL, BIM, BAD, and (anti-apoptotic) BAD phosphorylation, but *Sharpin^cpdm^* B cells showed more caspase 9 activation, which depends on cytochrome C release from the mitochondria and in turn activates caspase 3 (**Figure 7E** and **Supplementary Figure 4**). The caspase 9 activation started 2 h after stimulation and continued to increase until 48 h. At this time point, caspase 8 of the mitochondria-independent apoptosis pathway was also activated in *Sharpin^cpdm^* B cells, but not in *Sharpin^+/+^*B cells despite its activation at early time points, when caspase 9 was marginally activated (**Figure 7E** and **Supplementary Figure 4**). These, together with the rescue of the viability of *Sharpin^cpdm^* B cells by the inhibitor of caspase 8 or caspase 9 (**Figure 6D**), showed that the SHAPRIN deficiency resulted in the synchronized activation of these two principal initiator caspases, which together triggered the irreversible apoptosis process in *Sharpin^cpdm^* B cells.

The activation and function of caspase 8 are tightly regulated by a catalytically inactive homologous factor cFLIP, which includes the longer (cFLIP_L_) and shorter (cFLIP_R_) forms generated through alternative splicing of the *Cflar* transcript in mice, with cFLIP_L_ acting as either an inhibitor or a potentiator depending on its protein expression level while cFLIP_R_ acting exclusively as an inhibitor – both isoforms are unstable proteins whose concentrations determine the sensitivity of cells to mitochondria-independent apoptosis (40). In *Sharpin^+/+^*B cells, the kinetics of the caspase 8 activation, i.e., first induced as early as 2 h after stimulation, peaking at 24 h and then decreased to the pre-stimulation level at 48 h, was mirrored by the kinetics of the cleavage of the full-length cFLIP_L_ at Asp377 (equivalent to Asp376 of human cFLIP_L_) to generate p43-cFLIP_L_ and also by the kinetics of the expression of full-length or total cFLIP_L_, likely reflecting a feedback control (**Figures 7E, F**). By contrast, full-length cFLIP_L_ was continuously cleaved to yield p43-cFLIP_L_ in *Sharpin^cpdm^* B cells, with its protein level briefly induced after 2 h, starting to decrease at 6 h and diminished at 48 h, showing an inverse correlation with caspase 8 activation (**Figures 7E, F**). The reduced cFLIP_L_ protein expression in *Sharpin^cpdm^* B cells occurred despite normal *Cflar* gene transcription and was instead associated with decreased linear ubiquitination at the Met1 (linear M1-Ub) and increased polyubiquitination at Lys48 (K48 poly-Ub) of cFLIP_L_ (**Figures 7G, H**), consistent with the notion that the cFLIP_L_ protein level is controlled by proteasome degradation in a manner dependent on its K48 poly-Ub, which is catalyzed by the ITCH/AIP4 E3 ubiquitin ligase and was suggested to be hampered by linear M1-Ub of cFLIP_L_ (41, 42). The overall linear M1-Ub was modestly reduced in *Sharpin^cpdm^* B cells, likely reflecting the residual catalytic activities of the HOIP-HOIL sub-complex formed in the absence of SHARPIN. Finally, expression of XIAP, a potent inhibitor of the initiator caspase 7 as well as caspase 9 and caspase 3, was largely comparable in *Sharpin^+/+^*and *Sharpin^cpdm^* B cells (**Figure 7E** and **Supplementary Figure 4**), highlighting the specificity of SHARPIN regulation of cFLIP_L_.

Thus, SHARPIN deficiency modulates post-translational modifications of cFLIP_L_, abolishes the expression of this caspase 8 regulator and boosts caspase 8 activation, and synchronizes the activation of caspase 8 with that of caspase 9 in the intrinsic apoptosis pathway without affecting BCL2 family factors.

### SHARPIN Deficiency and HOIPIN-8 Synergize to Promote IL-21-Induced cFLIP_L_ Loss and Apoptosis

**Figure 8:**
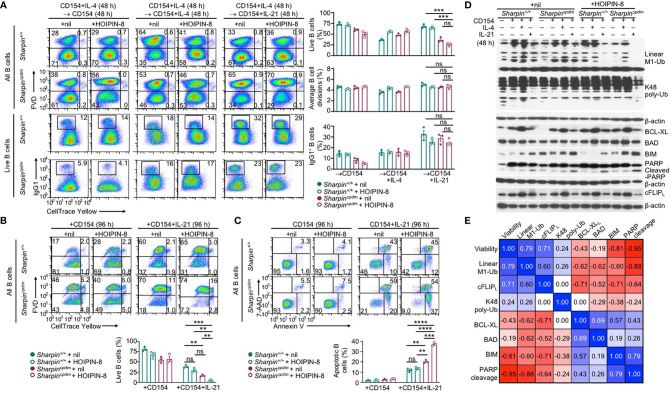
SHARPIN deficiency and HOIPIN-8 synergize to promote IL-21-induced cFLIP_L_ loss and apoptosis. **(A)** Flow cytometry analysis of survival, proliferation, and CSR to IgG1 in *Sharpin^+/+^* and *Sharpin^cpdm^* B cells stimulated first with CD154 and IL-4 for 48 h, washed, and then with CD154, alone or plus IL-4 or IL-21, for 48 h in the presence of nil or HOIPIN-8 (20 μM). **(B)** Flow cytometry analysis of the viability of *Sharpin^+/+^* and *Sharpin^cpdm^* B cells stimulated with CD154 or CD154 plus IL-21 for 96 h in the presence of nil or HOIPIN-8. **(C)** Flow cytometry analysis of apoptosis in *Sharpin^+/+^* and *Sharpin^cpdm^* B cells stimulated with CD154 or CD154 plus IL-21 for 96 h in the presence of nil or HOIPIN-8. **(D)** Immunoblotting analysis of linear M1-Ub, K48 poly-Ub, expression of apoptosis-related factors and PARP cleavage in *Sharpin^+/+^* and *Sharpin^cpdm^* B cells freshly isolated or stimulated, as indicated, for 24 h. Representative of two independent experiments. **(E)** Pair-wise correlation of different parameters, as indicated, in *Sharpin^+/+^* and *Sharpin^cpdm^* B cells stimulated with CD154 or CD154 plus IL-21 in the presence of nil or HOIPIN-8 for 96 h (to determine cell viability; data from were **B**) or 24 h (other parameters; data were from **D** and **Supplementary Figure 5D**). **p < 0.01; ***p < 0.001; ****p < 0.0001; t-test.

Like the genetic ablation of *Sharpin*, LUBAC inhibitor HOIPIN-8 hampered activation of NF-κB by αCD40 in B cells, starting at 50 μM (**Supplementary Figure 5A**). At 20 μM, HOIPIN-8 did not affect NF-κB activation and had no impact on proliferation, IL-4-directed CSR to IgG1 or IL-21-triggered plasma cell differentiation in CD154-stimulated wildtype B cells (**Supplementary Figure 5B**). Neither did it adversely affect the normal proliferation or CSR in *Sharpin^cpdm^* B cells (**Figure 8A**). Rather, HOIPIN-8 markedly increased the sensitivity of CD154-stimulated *Sharpin^cpdm^* B cells to IL-21-induced apoptosis and cell death in a dose-dependent manner and virtually abrogated the viability of these cells after 96 h of culture, in contrast to its marginal impact on the viability of *Sharpin^+/+^*B cells (**Figures 8B, C** and **Supplementary Figure 5C**).

In *Sharpin^+/+^*B cells, total linear M1-Ub was induced upon stimulation by CD154, but returned to the basal level when IL-21 was added – IL-4 had no such dampening effect (**Figure 8D**). Treatment with HOIPIN-8 (at 20 μM) and SHARPIN deficiency each reduced linear M1-Ub, but together abrogated it in CD154 and IL-21-stimulated B cells (**Figure 8D** and **Supplementary Figure 5D**), likely due to the partial and full sensitivity of the HOIP-HOIL-SHARPIN complex and HOIP-HOIL sub-complex, respectively, to HOIPIN-8 at sub-optimal concentrations. Combining the HOIPIN-8 treatment and SHARPIN deficiency also decreased, by about 75%, linear M1-Ub induced by CD154 or CD154 plus IL-4, while each alone led to a 30-40% reduction (**Figure 8D** and **Supplementary Figure 5D**). Finally, the level of linear M1-Ub was positively correlated with the viability of B cells stimulated by CD154 or CD154 plus IL-21 (**Figure 8E** and **Supplementary Figure 5E**).

Unlike linear M1-Ub, the total K48 poly-Ub level was barely inducible; nor was it impaired by SHARPIN deficiency or the HOIPIN-8 treatment, which did not change the expression of anti-apoptotic factor BCL-XL and pro-apoptotic factor BAD – CD154 and IL-21-stimulated *Sharpin^cpdm^* B cells did show 35% reduction of K48 poly-Ub after HOIPIN-8 treatment (**Figure 8D** and **Supplementary Figures 5D, E**). Accordingly, the levels of K48 poly-Ub level, BCL-XL, and BAD showed weak or no correlation with B cell viability (**Figure 8E**). By contrast, the expression of pro-apoptotic BIM and the level of the PARP cleavage, which is mediated by effector caspases during apoptosis, were synergistically elevated by the SHARPIN deficiency and HOIPIN-8 treatment, with both showing a strong negative correlation with the B cell viability (**Figures 8D, E** and **Supplementary Figure 5D**). Finally, the expression of the full-length cFLIP_L_ protein closely tracked the level of linear M1-Ub, which was severely reduced in HOIPIN-8-treated CD154 and IL-21-stimulated *Sharpin^cpdm^* B cells, and positively correlated with the B cell viability (**Figures 8D, E** and **Supplementary Figure 5D, 5E**).

In summary, the function of LUBAC in B cells is controlled by the availability of SHARPIN and the activity of HOIP. LUBAC, in turn, regulates the protein level of cFLIP_L_ and critically promotes the survival of B cells stimulated with CD154 in the presence of IL-21.

## Discussion

This study has described the B cell-intrinsic role of LUBAC in promoting B cell survival from IL-21-triggered apoptosis, as relevant to the GC reaction, positive selection, and production of class-switched high-affinity Abs. Our data, as stemming from experiments involving B-*Sharpin^cpdm^* and *Cd45.1^+^-Sharpin^+/+^*/*Cd45.2^+^-Sharpin^cpdm^* chimera mice, have significantly extended findings from a previous report, which showed that *mb1^+/cre^Hoip^fl/fl^* mice displayed much reduced Ab responses, including those to T-independent Ags, but did not identify the *in vivo* defect of *mb1^+/cre^ Hoip^fl/fl^* B cells underlying such impairment (31, 43). By contrast, our extensive analyses of B cell proliferation, survival, differentiation (CSR and plasma cell differentiation) as well as SHM and enrichment of a mutation that augments the affinity of BCRs have identified a key defect of SHARPIN-deficient B cells, i.e., reduced survival within GCs, that would provide a parsimonious explanation for the impaired T-dependent Ab response in mice with B cell-specific ablation of LUBAC. Confirming a causative role of the defective *Sharpin^cpdm^* GC B cell survival in this process would require generating mice with simultaneous *Sharpin* (or *Hoip*) deletion and enforced expression of an apoptosis inhibitor, such as a dominant negative mutant of effector caspase 3 (44), specifically in GC B cells (e.g., through *Cγ1-cre* or *Aicda-cre*) – genetic alterations of a single factor in the mitochondria-dependent intrinsic pathway or the mitochondria-independent pathway would be less likely to maintain the full GC B cell viability. The viability of GC B cells in B-*Sharpin^cpdm^* mice may also be restored by caspase 3 inhibitor Z-VAD-FMK (or possibly Z-IETD-FMK combined with Z-LEHD-FMK), but further compromised by HOIPIN-8. Finally, *Sharpin^cpdm^* B cells were also defective in survival upon TLR ligand stimulation, likely explaining the reduced production of IgG3 and IgG2b Abs.

**Figure 9:**
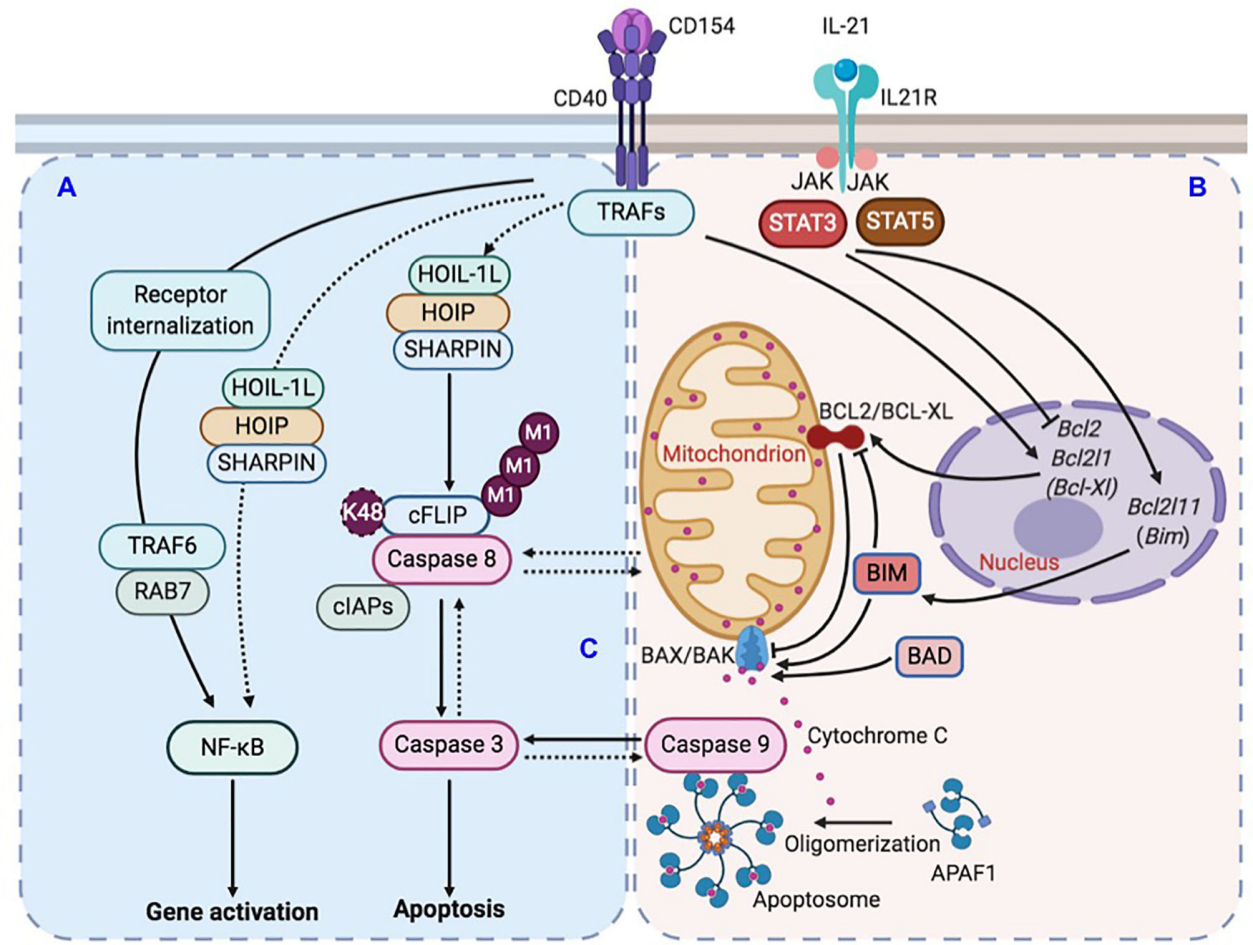
Illustration of LUBAC-mediated suppression of IL-21-induced apoptosis in CD154-stimulated B cells. **(A)** CD40 activation in B cells upregulates LUBAC-catalyzed linear M1-Ub, including that of cFLIP, which leads to cFLIP stabilization and inhibition of caspase 8 activation. LUBAC plays a minor role to the major role of RAB7 in CD40-triggered NF-κB activation in B cells. **(B)** CD40 activation also induces the expression of anti-apoptotic factor BCL2 and BCL-XL. IL-21, however, dampens such induction and also upregulates expression of pro-apoptotic factor BIM. The combined effects of BCL2/BCL-XL downregulation and BIM upregulation would activate pro-apoptotic BAX/BAK complex to increase the mitochondria permeability and cytochrome C release into the cytoplasm to activate caspase 9. **(C)** In the absence of LUBAC, caspase 8 activation would amplify a putative caspase network, within which multiple caspases, including caspase 9 and caspase 3, would be activated in a synchronized manner due to positive-feedback loops, leading to the irreversible apoptosis process.

Shortly after the discovery of IL-21 and its crucial role in plasma cell differentiation, the potent B cell killing activity of this cytokine was recognized (10, 45, 46), although how this killing activity is circumvented in GCs and its relevance to the T-dependent Ab response remain unaddressed. This was in part due to the difficulty of untangling its effect from the important role of IL-21 in promoting the proliferation of B cells activated by sub-optimal CD40 signals (e.g., as initiated by αCD40 or low doses of CD154). As shown here, the SHARPIN deficiency decoupled the survival from the proliferation/differentiation of GC B cells *in vivo* and B cells activated by CD154 and IL-21 *in vitro*, thereby providing an opportunity to reveal the role of IL-21-triggered B cell death in the GC reaction, including the positive selection of high-affinity BCR mutants, and underlying mechanisms. CD40-activated B cells would need at least two mechanisms to survive the assault by IL-21, i.e., potent CD40 signals that induce anti-apoptotic BCL2 and BCL-XL to counter the effect of IL-21 in activating the mitochondria-dependent intrinsic apoptosis pathway (**Figure 9B**) as well as LUBAC that inhibits caspase 8, thereby preventing synchronized caspase 8 and -9 activation, full mobilization of caspase 3 and irreversible apoptosis (**Figure 9A**). The requirement for LUBAC would be important to preserve all GC B cells, irrespective of the affinity of their BCRs, particularly when such B cells are at the early phase of the iterative proliferation and selection process, i.e., completing fewer divisions. The requirement for strong CD40 signals would be consistent with the notion that GC B cell clones are differentially selected based on the quality and quantity of their contacts with Tfh cells due to their differences in BCR affinity and, therefore, the Ag uptake and presentation to Tfh cells. However, more contacts would also lead to more exposure to IL-21 produced by the same Tfh cells and, therefore, a higher threshold of B cell survival. Thus, the entwined and counteracting effects of CD154 and IL-21 would make them the prime candidate for enforcing a continuous positive selection process underpinned by the co-evolution (i.e., co-upregulation) of the death-inducing signals and the survival signal after each round of the selection. Such co-evolution would explain the stepwise improvement of the Ab affinity, a crucial aspect of the positive selection that had, however, been largely unexplored. Notably, Tfh cells were largely normal in B-*Sharpin^cpdm^*mice despite the reduced GC reaction, a phenotype similar to that of IL-21- and IL-21R-deficient mice (11, 47), perhaps reflecting a compensatory role of CD154 plus IL-4-activated B cells in the engagement and maintenance of Tfh cells – such B cells would not be affected by the *Sharpin* deficiency in their proliferation, survival, CSR or plasma cell differentiation, as shown here. Complementing the role of Tfh cells, elevated signaling from high-affinity BCRs may also be involved in maintaining GC B cells despite being downregulated in these cells (48, 49), but unlikely to induce B cell death or involved in the stepwise affinity maturation process. Finally, the *Sharpin^cpdm^* B cells activated *in vitro* showed normal expression of *Myc* and *mTORC1* (our RNA-Seq data, not shown), which promote the expansion of selected B cell clones (50–53), suggesting that the defective positive selection of B-*Sharpin^cpdm^* mice was not due to reduced proliferation of high-affinity clones.

The high-throughput V_186.2_-DJ_H_ sequences reported here could be used to construct “lineage trees”. Each tree would be rooted in a distinct progenitor, acquiring a dominant branch stemming from a high-affinity founder sub-clone (e.g., that carrying the G_98_→T_98_ mutation) and further branched with new rounds of SHM, thereby opening a window for observation into the positive selection process and its impairment in *Sharpin^cpdm^* B cells, as lack of robust positive selection would result in overall less complex lineage trees. In addition, the exclusive occurrence of G_98_→T_98_ in V_186.2_-DJ_H_-Cγ1, but not V_186.2_-DJ_H_-Cμ, provides additional, albeit indirect, evidence for the notion that IL-4-directed CSR to IgG1 preceded IL-21-mediated development of GCs, within which peak SHM and positive selection unfold (16). Also along this line, pre-activation of *Sharpin^+/+^* or *Sharpin^cpdm^* B cells with CD154 plus IL-4 did not make them resistant to death induced by IL-21, the production of which lags the IL-4 production by NKT cells and Th2 cells before the GC reaction (15, 54), indicating that sensitivity to IL-21-triggered death is an important feature intrinsic to GC B cells. By contrast, pre-activation by TLR ligands promoted the survival of B cells activated by sub-optimal CD40 signaling (e.g., as initiated by αCD40) and IL-21, likely mediating their differentiation into IL-27-producing B cells (55). Finally, the ability of strong BCR crosslinking by αIgD/dex to endow *Sharpin^cpdm^* B cells resistance to IL-21 suggests that BCR signaling partially contributed to the GC B cell survival in B-*Sharpin^cpdm^* mice, possibly by upregulating MYC (56).

As previously shown (45, 46) and further extended here, IL-21 induced apoptosis through the intrinsic pathway by modulating the expression of the BCL2 family of pro-apoptotic and anti-apoptotic members that control the mitochondria membrane permeability and cytochrome C release for caspase 9 activation. Without a death domain (DD), the IL-21 receptor is unable to recruit DD-containing adaptors that initiate the death effector domain (DED)-dependent caspase 8 activation. CD40 has no DD either, in contrast to FAS (and other TNF receptor superfamily members, such as TNFR and TRAIL), raising the possibility that, in CD154 and IL-21-stimulated B cells, caspase 8 was activated by a receptor-independent apoptosis process, e.g., through a macromolecular complex nucleated by the auto-aggregation of DD- and DED-containing adaptor FADD followed by pro-caspase 8 recruitment and self-activation, as occurring in cancer cells (57). Such caspase 8 activation would be subsequently suppressed in *Sharpin^+/+^* B cells due to upregulation of cFLIP, but not in *Sharpin^cpdm^* B cells, in which cFLIP level was significantly reduced, as shown here. Nevertheless, the full and sustained activation of caspase 8 might require caspase 9 activation by IL-21 and ultimately caspase 3, perhaps within a recently suggested caspase network (58) (**Figure 9C**). Within such a network, positive feedback loops (e.g., those among the initiator caspases and between the initiator and effector caspases) would synchronize the activation of multiple caspases, as likely underpinned by caspase cleavage-mediated inactivation of anti-apoptosis factors and activation of pro-apoptotic factors, including caspase 8 activation of BID to trigger caspase 9 (59). The rescue of the viability of IL-21-stimulated B cells by enforced BCL2 overexpression or deficiency in BIM (45, 46) emphasizes the role of the intrinsic apoptosis pathway but does not rule out its collaboration with activated caspase 8 to fully activate caspase 3. Finally, IL-21 readily induced apoptosis in B cells deficient in FAS, TNFRI, or TNFRII (46), showing that IL-21 did not cause cell death indirectly by inducing these death receptors.

Although SHARPIN was originally identified as a key LUBAC component for NF-κB activation by αCD40 in Ramos B lymphoma cells (23, 24), the LUBAC pathway did not play a major role in NF-κB activation in CD154-stimulated primary B cells, which rather use CD40 endocytosis and the RAB7-dependent endosomal pathway to activate NF-κB (39). Instead, CD154 upregulated, in addition to BCL2 and BCL-XL, and Linear M1-Ub (**Figures 9A, B**), which is exclusively catalyzed by different forms of complexes containing HOIP, HOIL-1, and/or SHARPIN and, as shown here, had a strong a positive correlation with the viability of B cells stimulated with CD154 plus different cytokine combinations. This correlation was unveiled by the partial defect of *Sharpin^cpdm^* B cells in catalyzing Linear M1-Ub and the use of a sub-optimal dose of HOIPIN-8. Furthermore, despite the strong correlation among the B-cell viability, Linear M1-Ub, and c-FLIP_L_ levels, a definitive role of linear ubiquitinated c-FLIP_L_ in maintaining the B cell viability remains to be proven, possibly by the specific disruption of interaction of c-FLIP_L_ with LUBAC and, conversely, the rescue of the viability of HOIPIN-8-treated *Sharpin^cpdm^* B cells with enforced expression of c-FLIP_L_, preferably a form that carries a K48 mutation and, therefore, is resistant to K48 polyubiquitination and proteasome degradation. Although it has long been recognized that cFLIP plays a role in the activation and survival of immune cells (60–65), including T cells and B cells, exactly how its level (a limiting factor in the anti-apoptosis activity) in immune cells is regulated, including by LUBAC, remains to be defined. As cFLIP also controls the pivoting between apoptosis and necroptosis, either through receptor-induced RIP kinase or receptor-independent assembly of riptosomes, whether and how cFLIP regulates necroptosis to influence the outcome of B cell viability and GC reaction needs to be explored, particularly in light of recent findings showing the unexpected role of caspase 9 in inhibiting necroptosis to promote GC B cell maintenance (66). Finally, cFLIP downregulation sensitized B lymphoma cells to TRAIL-induced apoptosis and breast cancer cells to ligand-independent but FADD-, caspase 8- and caspase 9-dependent apoptosis (65, 67), suggesting that HOIPIN-8, which downregulated cFLIP expression, could potentially be developed into therapeutics for B cell lymphoma as well as systemic lupus, particularly the disease caused by heightened apoptosis threshold due to lack of FAS.

## Data Availability Statement

The datasets presented in this study can be found in online repositories. The names of the repository/repositories and accession number(s) can be found below: NCBI SRA data PRJNA704065, BioSample accessions: SAMN18029793, SAMN18029794.

## Ethics Statement

The animal study was reviewed and approved by The Institutional Animal Care and Use Committee (IACUC) of UTHSCSA.

## Author Contributions

Conceptualization (JW, HY, and ZX), investigation (JW, CR, HY, and ZX), visualization (JW, TL, HZ, and HY), funding acquisition (ZX), and supervision (HY and ZX). All authors contributed to the article and approved the submitted version.

## Funding

This work was supported by NIH AI 124172, AI 131034, AI 135599, AI 153506, and DOD BC170448 grants. The UTHSCSA Flow Cytometry Core facility is supported by NIH P30 CA054174 and UL1 TR001120, and Genome Sequencing Facility supported by NIH P30 CA054174, S10 OD021805, and CPRIT Core RP160732 grants.

## Conflict of Interest

The authors declare that the research was conducted in the absence of any commercial or financial relationships that could be construed as a potential conflict of interest.

## Supplementary Material

Supplementary Figure 1–5

Supplementary Table 1 Antibodies and reagents.

Supplementary Table 2 Oligonucleotide sequences.

## Abbreviations

AID, Activation induced cytidine deaminase; Ab, antibody; Ag, antigen; ASCs, antibody secreting cells; mAb, monoclonal antibody; BCR, B cell receptor; CDR, complementarity determining region; FADD-like IL-1β-converting enzyme -inhibitory protein; cFLIP, cellular FLICE; CSR, class switch DNA recombination; *Sharpin^cpdm^*, *Sharpin* chronic proliferative dermatitis mutation; FR, framework region; GC, germinal center; HOIL-1, Heme-Oxidized IRP2 Ubiquitin Ligase 1; HOIP, HOIL-1-interacting protein; HOIPIN-8, HOIP inhibitor 8; IgH, immunoglobulin heavy chain; LUBAC, linear ubiquitin chain assembly complex; NF-κB, nuclear factor κB; SHARPIN, SHANK-associated RH domain interacting protein; SHM, somatic hypermutation; Tfh cells, T follicular helper cells; XIAP, X-linked inhibitor of apoptosis.
