# Supplementary material for: LUBAC Suppresses IL-21-Induced Apoptosis in CD40-Activated Murine B Cells and Promotes Germinal Center B Cell Survival and the T-Dependent Antibody Response
Source: Front Immunol. 2021 Apr 19;12:658048. doi: 10.3389/fimmu.2021.658048 (PMC8089397; doi:10.3389/fimmu.2021.658048)
Supplement: Supplementary Figure 1–5 [file DataSheet_1.pdf]

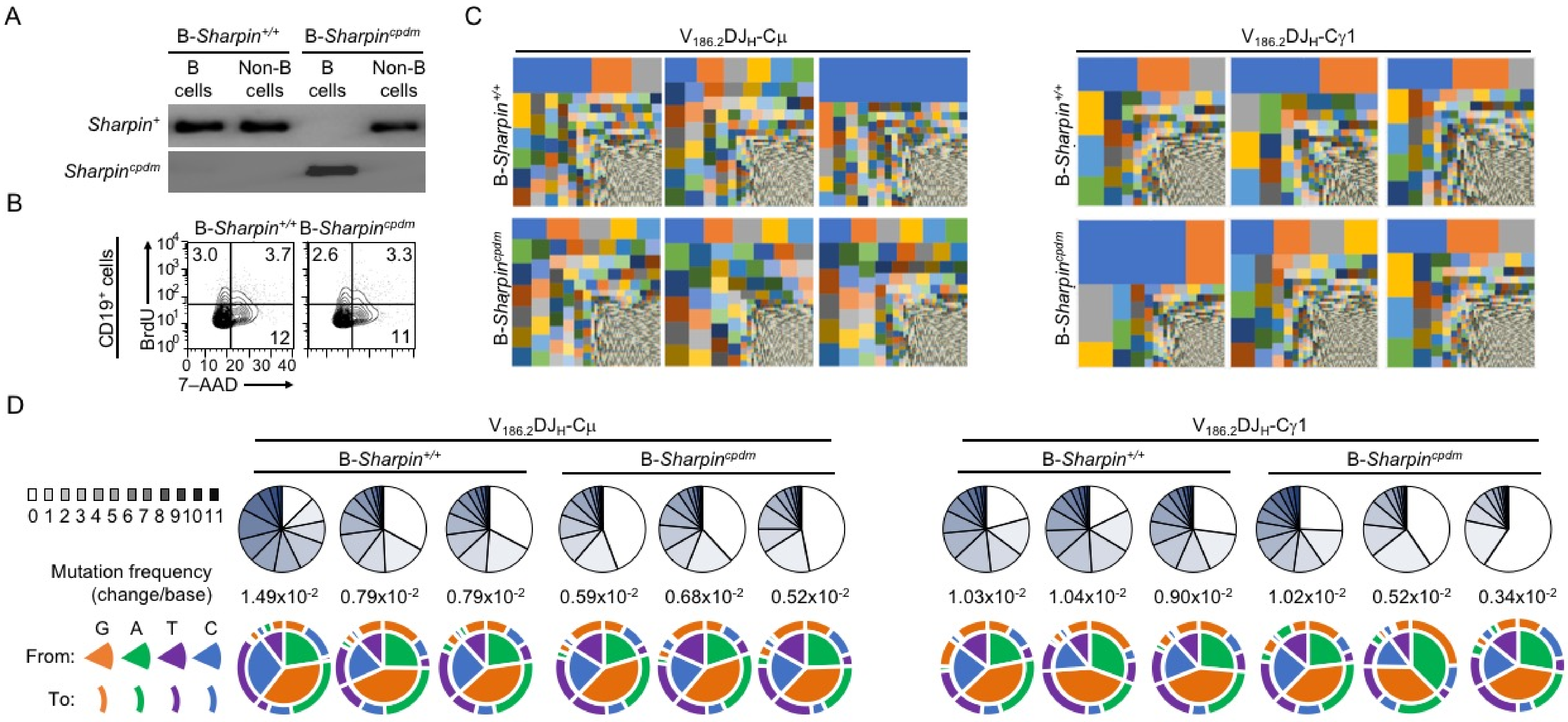

SUPPLEMENTAL FIGURE 1 | (**A**) Genotyping of B cells, as isolated by negative selection, and non-B cells, which were those that bound mAbs and magnetic beads during the negative selection, from the spleen of B-*Sharpin*$^{+/+}$ and B-*Sharpin*^{cpdm} mice. (**B**) Flow cytometry analysis of B cell proliferation (by BrdU incorporation) in the spleen of B-*Sharpin*$^{+/+}$ or B-*Sharpin*^{cpdm} mice 14 d after immunization with NP-CGG/alum. (**C**) Treemap charts depicting B cell clones identified by unique CDR3 sequences in the V_H region of $V_{186.2}DJ_H$-Cμ and $V_{186.2}DJ_H$-Cγ1 in three B-*Sharpin*$^{+/+}$ mice and three B-*Sharpin*^{cpdm} mice 14 d after NP-CGG/alum immunization. Each rectangle represents a unique clone and the size of each rectangle depicts the relative abundance of the clone within the total population. (**D**) Proportions of $V_{186.2}DJ_H$-Cμ and $V_{186.2}DJ_H$-Cγ1 clones carrying given numbers of point-mutations in $V_{186.2}$ region in three B-*Sharpin*$^{+/+}$ mice and three B-*Sharpin*^{cpdm} mice 14 d after NP-CGG/alum immunization, as depicted by pie chart slices (top row; sequences with over 12 point-mutations were excluded from analysis), the overall mutation frequencies (middle), and the nature of such point-mutations, as depicted by concentric pie chart slices and rings (bottom row).

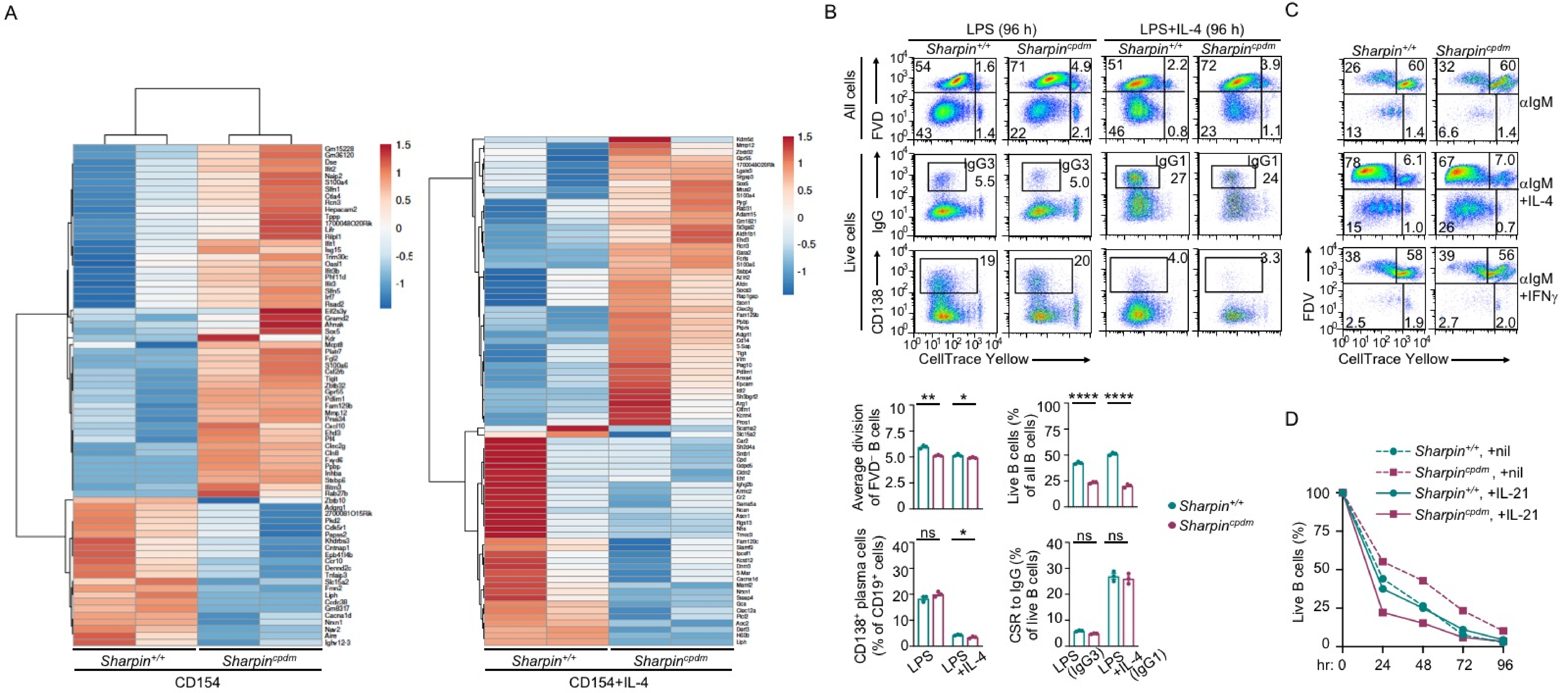

SUPPLEMENTAL FIGURE 2 | (**A**) Heatmap of differentially expressed genes (DEGs) between *Sharpin*$^{+/+}$ and *Sharpin*^{cpdm} B cells stimulated with CD154 or CD154 plus IL-4 for 24 h. Data are from two independent B cell stimulation and RNA-Seq experiments. (**B**) Flow cytometry analysis of proliferation, cell survival, CSR to IgG1 or IgG3 and plasma cell differentiation in *Sharpin*$^{+/+}$ and *Sharpin*^{cpdm} B cells after stimulated with LPS or LPS plus IL-4 for 96 h. (**C**) Flow cytometry analysis of proliferation and survival of *Sharpin*$^{+/+}$ and *Sharpin*^{cpdm} B cells stimulated, as indicated, for 96 h. (**D**) Flow cytometry analysis of proliferation and survival of *Sharpin*$^{+/+}$ and *Sharpin*^{cpdm} B cells stimulated with nil or IL-21 for different periods of time, as indicated. (**C**, **D**) Representative of three independent experiments. * $p<0.05$; ** $p<0.01$; **** $p<0.0001$; t-test.

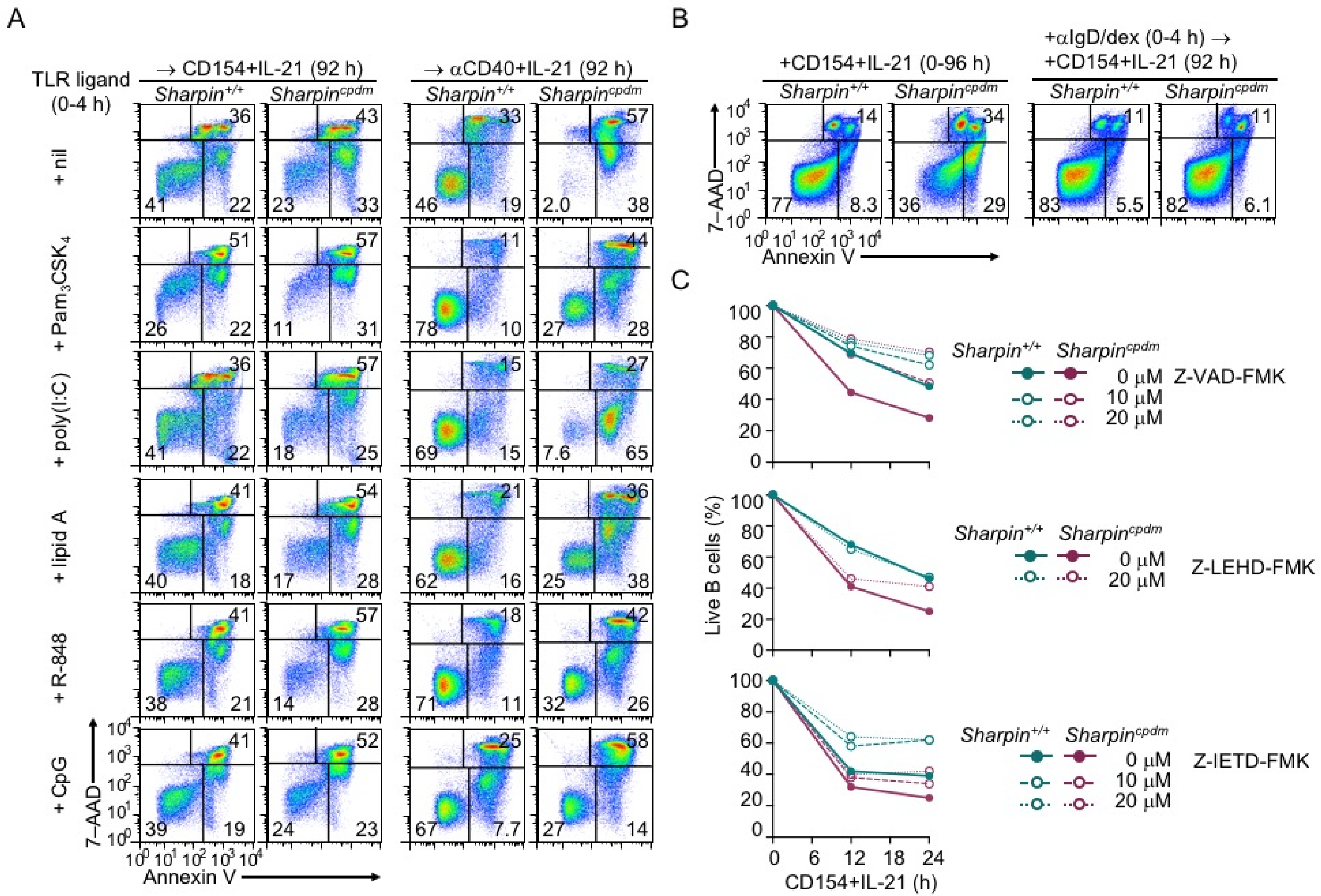

SUPPLEMENTAL FIGURE 3 | Flow cytometry analysis of apoptosis and necrosis in *Sharpin*$^{+/+}$ and *Sharpin*^{cpdm} B cells primed with TLR ligands (as indicated, **A**) or αIgD/dex (**B**) for 4 h and then stimulated with CD154 or αCD40 plus IL-21 for 92 h. (**C**) Flow cytometry analysis of viability of *Sharpin*$^{+/+}$ and *Sharpin*^{cpdm} B cells stimulated with CD154 plus IL-21 in the presence of pan-caspase inhibitor Z-VAD-FMK, caspase 9- specific inhibitor Z-LEHD-FMK or caspase 8-specific inhibitor Z-IETD-FMK at different doses, as indicated, for 12 h or 24 h.

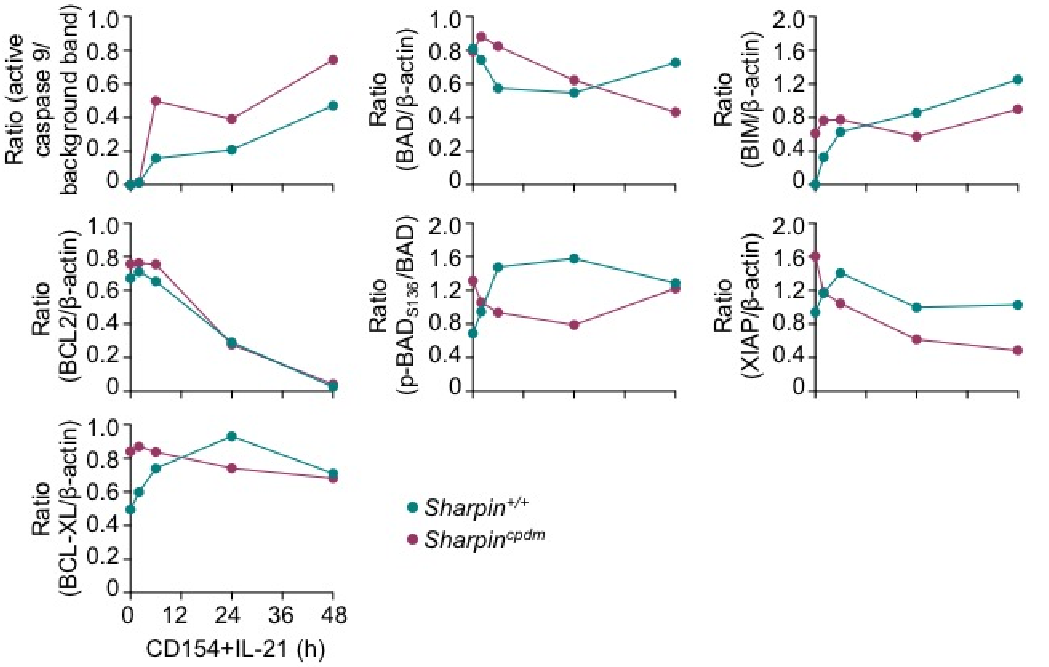

SUPPLEMENTAL FIGURE 4 | Immunoblotting analysis and quantified signals of caspase 9 activation, BCL2 and BCL-XL expression, BAD expression and phosphorylation as well as expression of BIM and XIAP in *Sharpin*$^{+/+}$ and *Sharpin*^{cpdm} B cells stimulated with CD154 and IL-21 for different periods of time, as indicated. Data are from Figure 7E.

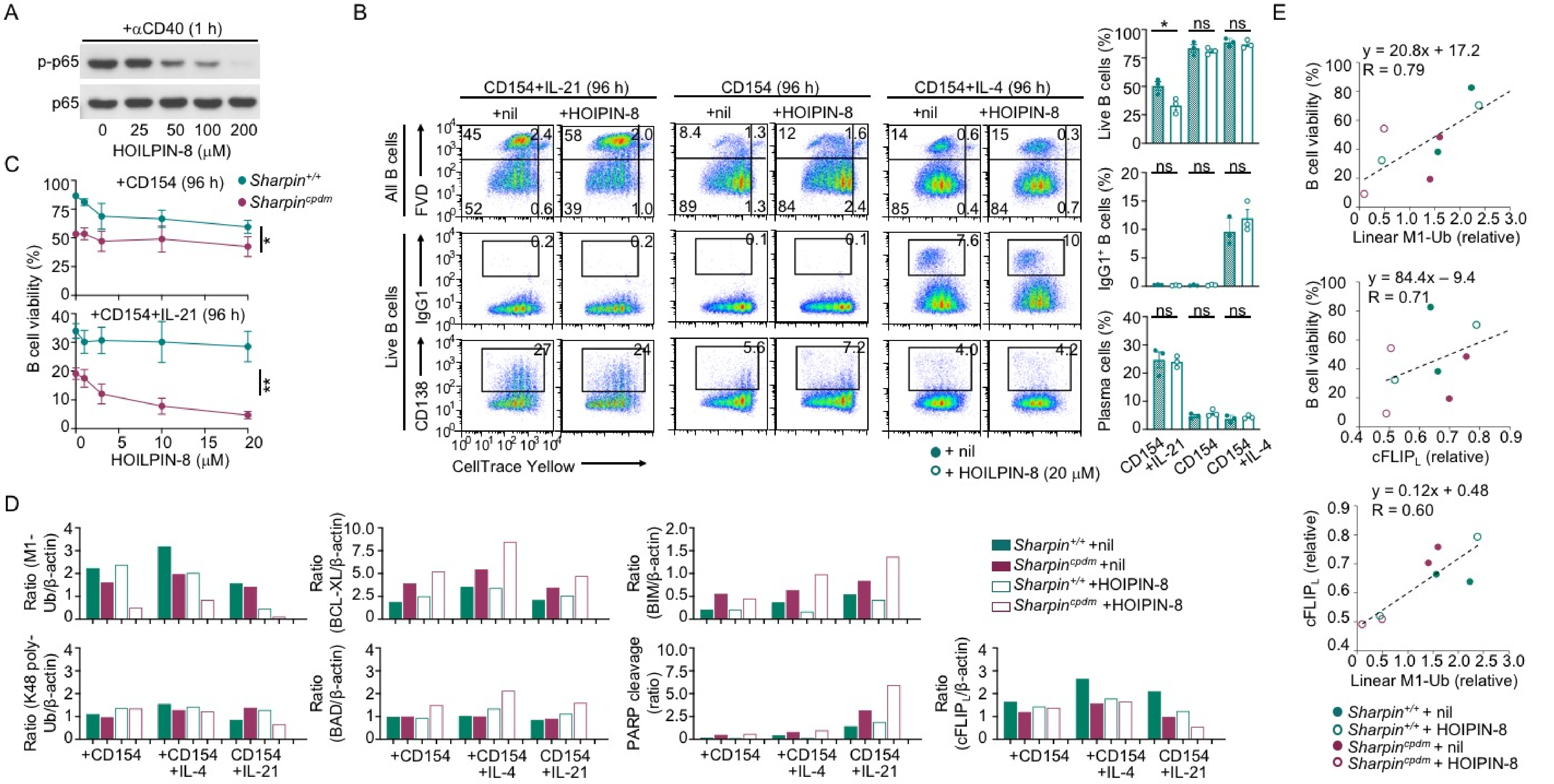

SUPPLEMENTAL FIGURE 5 | (**A**) Immunoblotting of phosphorylated p65 and total p65 protein levels in C57 B cells after stimulation by αCD40 for 1 h in the presence of different concentrations of HOIPIN-8, as indicated. Representative of two independent experiments. (**B**) Flow cytometry analysis of proliferation, survival, CSR to IgG1 and plasma cell differentiation of C57 B cells stimulated, as indicated, for 96 h in the absence or presence of HOIPIN-8 (*, <0.05; t-test). (**C**) Flow cytometry analysis of the viability of *Sharpin*$^{+/+}$ and *Sharpin*^{cpdm} B cells stimulated with CD154 or CD154 plus IL-21 for 96 h in the presence of different concentrations of HOIPIN-8 (triplicates; *, <0.05; **, <0.01; t-test). (**D**) Quantified signals of linear M1-Ub, K48 poly-Ub, expression of apoptosis factors and PARP cleavage, as determined by immunoblotting analysis, in *Sharpin*$^{+/+}$ and *Sharpin*^{cpdm} B cells stimulated with CD154 and IL-21 for 48 h in the absence or presence of HOIPIN-8. Data are from Figure 8E. (**E**) Correlation analysis of *Sharpin*$^{+/+}$ and *Sharpin*^{cpdm} B cell viability (after 96 h of stimulation by CD154 or CD154 plus IL-21 in the absence or presence of HOIPIN-8; data are from Figure 8B) with the level of linear M1-Ub and the cFLIP$_L$ protein level as well as that of the cFLIP$_L$ protein level with linear M1-Ub and K48 poly-Ub (all after 48 h of stimulation by CD154 or CD154 plus IL-21; data are from Figure 8D and Supplemental 5D).
