# Supplementary material for: LUBAC Suppresses IL-21-Induced Apoptosis in CD40-Activated Murine B Cells and Promotes Germinal Center B Cell Survival and the T-Dependent Antibody Response
Source: Front Immunol. 2021 Apr 19;12:658048. doi: 10.3389/fimmu.2021.658048 (PMC8089397; doi:10.3389/fimmu.2021.658048)
Supplement: Supplementary Table 1 — Antibodies and reagents. [file DataSheet_2.pdf]

Supplemental Table 1. Antibodies and reagents.

Flow Cytometry		
αmuCD19-BV421™	BD Biosciences	Cat. # 562701 (Clone 1D3)
αmuCD19-APC	TONBO Biosciences	Cat. # 20-0193 (Clone 1D3)
αmuB220-FITC	BioLegend	Cat. # 103206 (Clone RA3-6B2)
αmuCD3-Pacific Blue™	BioLegend	Cat. # 100214 (Clone 17A2)
αmuCD3-APC-Cy7	BioLegend	Cat. # 100221 (Clone 17A2)
αmuCD4-APC	BioLegend	Cat. # 100412 (Clone GK1.5)
αmuCD4-PerCP-Cy5.5	eBioscience	Cat. # 45-0042-82 (Clone RM4-5)
αmuCD8b-PE	eBioscience	Cat. # 12-0083-81 (Clone H35-17.2)
αmuCD11b-APC	BioLegend	Cat. # 101212 (Clone M1/70)
αmuCD11c-eFuor450	eBioscience	Cat. # 48-0114-82 (Clone N418)
αmuCD11c-APC-Cy7	BioLegend	Cat. # 117323 (Clone N418)
αmuIgM-PE	BioLegend	Cat. # 406508 (Clone RMM-1)
αmuIgD-APC	BioLegend	Cat. # 405714 (Clone 11-26c.2a)
αmuIgG1-APC	BioLegend	Cat. # 406609 (Clone RMG1-1)
αmuIgG3-BV421™	BD Biosciences	Cat. # 565808 (Clone R40-82)
αmuc-Kit-PE-Cy7	BioLegend	Cat. # 105813 (Clone 2B8)
αmuCXCR5-APC	BioLegend	Cat. # 145506 (Clone L138D7)
αmuCD23-PE	BioLegend	Cat. # 101607 (Clone B3B4)
αmuCD45-BV650™	BioLegend	Cat. # 103151 (Clone 30-F11)
αmuCD45-PerCP	BioLegend	Cat. # 103130 (Clone 30-F11)
αmuCD45.1-BV510™	BioLegend	Cat. # 110471 (Clone A20)
αmuCD45.2-PE-Cy7	BioLegend	Cat. # 109830 (Clone 104)
αmuAA4.1-APC	BioLegend	Cat. # 136509 (Clone AA4.1)
αmuFAS-BV510™	BD Biosciences	Cat. # 563646 (Clone Jo2)
αmuGL-7-PE	BioLegend	Cat. # 144607 (Clone GL7)
αmuGL-7-PerCP-Cy5.5	BioLegend	Cat. # 144609 (Clone GL7)
αmuGr-1- Pacific Blue™	BioLegend	Cat. # 108430 (Clone RB6-8C5)
αmuSca-1-PerCP	BioLegend	Cat. # 108121 (Clone D7)
αmuLineage-BV421™	BioLegend	Cat. # 133311
αmuPD-1-FITC	eBioscience	Cat. # 11-9985-85 (Clone J43)
αCleaved caspase 3-PE	BD Biosciences	Cat. # 561011 (Clone C92-605)
αBrdU-APC	Biosearch Technologies	Cat. # 339808 (Clone Bu20a)
Immunofluorescence staining		
αmuB220-FITC	BioLegend	Cat. # 103206 (Clone RA3-6B2)
αmuGL7-PerCP-Cy5.5	BioLegend	Cat. # 144609 (Clone GL7)
ELISA/ELISPOT		
αmuIgM-UNLB	SouthernBiotech	Cat. # 1020-01
αmuIgG-UNLB	SouthernBiotech	Cat. # 1030-01

αmuIgA-UNLB	SouthernBiotech	Cat. # 1040-01
αmuIgM-biotin	SouthernBiotech	Cat. # 1020-08
αmuIgG1-biotin	SouthernBiotech	Cat. # 1070-08
αmuIgG2a-biotin	SouthernBiotech	Cat. # 1080-08
αmuIgG2b-biotin	SouthernBiotech	Cat. # 1090-08
αmuIgG3-biotin	SouthernBiotech	Cat. # 1100-08
αmuIgA-biotin	SouthernBiotech	Cat. # 1040-08
NP_7-BSA	Biosearch Technologies	Cat. # N-5050L-10
NP_{34}-BSA	Biosearch Technologies	Cat. # N-5050H-10
Other flow cytometry reagents		
Fixable Viability Dye eFlour™ 780	eBioscience	Cat. # 65-0865-14
Ghost Dye™ Red 710	TONBO Biosciences	Cat. # 13-0871-T500
7–Aminoactinomycin D (7–AAD)	Sigma Aldrich	Cat. # A9400
Immunoblotting		
αM1 linear ubiquitin	Millipore-Sigma	Cat. # MABS451 (Clone LUB9)
αK48-linkage Polyubiquitin	Cell Signaling Technology	Cat. # 12805
αUbiquitin	Cell Signaling Technology	Cat. # 3933
αCaspase-3 (D3R6Y)	Cell Signaling Technology	Cat. # 14220
αCleaved Caspase-3 (Asp175)	Cell Signaling Technology	Cat. # 9661T
αCleaved Caspase-9 (Asp353)	Cell Signaling Technology	Cat. # 9509T
αBIM (C34C5)	Cell Signaling Technology	Cat. # 2933T
αBCL-2 (D17C4)	Cell Signaling Technology	Cat. # 3498T
αCaspase-8 (1C12)	Cell Signaling Technology	Cat. # 9746
αCaspase-9 (C9)	Cell Signaling Technology	Cat. # 9508
αPARP	Cell Signaling Technology	Cat. # 9542
αcFLIP (D5J1E)	Cell Signaling Technology	Cat. # 56343
αBCL-XL (54H6)	Cell Signaling Technology	Cat. # 2764T
αBAX (D3R2M)	Cell Signaling Technology	Cat. # 14796S
αBAD (D24A9)	Cell Signaling Technology	Cat. # 9239
αPhospho-BAD (Ser136)	Cell Signaling Technology	Cat. # 4366
αCytochrome C (D18C7)	Cell Signaling Technology	Cat. # 11940
αPhospho-NF-κB p65 (Ser536)	Cell Signaling Technology	Cat. # 3033
αNF-κB p65 (D14E12)	Cell Signaling Technology	Cat. # 8242
αXIAP	Cell Signaling Technology	Cat. # 2042
αPhospho-STAT3 (Tyr705)	Cell Signaling Technology	Cat. # 9145
α Phospho-STAT5 (Tyr694)	Cell Signaling Technology	Cat. # 4322
αSTAT3 (79D7)	Cell Signaling Technology	Cat. # 4904
αSTAT5 (D2O6Y)	Cell Signaling Technology	Cat. # 94205
α-β-actin (13E5)	Cell Signaling Technology	Cat. # 4970S

Supplemental Table 2. Oligonucleotide sequences.

qRT-PCR		
Gene	Forward primer	Reverse primer
Aicda	5’-AGAAAGTCACGCTGGAGACC-3’	5’-CTCCTCTTCACCACGTAGCA-3’
Prdm1	5’-TTCTCTTGGAAAAACGTGTGGG-3’	5’-GGAGCCGGAGCTAGACTTG-3’
Iγ1-Cγ1	5’-TCGAGAAGCCTGAGGAATGTG-3’	5’-ATGGAGTTAGTTTGGGCAGCA-3’
Iγ2a-Cγ2a	5’-GCTGATGTACCTACCGAGAGA-3’	5’-GCTGGGCCAGGTGTTCGAGGTT-3’
Cflar	5’-GCTCCAGAATGGGCGAAGTAA-3’	5’-ACGGATGTGCGGAGGTAAAAA-3’
Bcl2	5’-ATGCCTTTGTGGAACTATATGGC-3’	5’-GGTATGCACCCAGAGTGATGC-3’
Bcl2l1	5’-GACAAGGAGATGCAGGTATTGG-3’	5’-TCCCGTAGAGATCCACAAAAGT-3’
Mcl1	5’-AAAGGCGGCTGCATAAGTC-3’	5’-TGGCGGTATAGGTCGTCCTC-3’
Bcl2l11	5’-CCCGGAGATACGGATTGCAC-3’	5’-GCCTCGCGGTAATCATTTGC-3’
Bad	5’-AAGTCCGATCCCGGAATCC-3’	5’-GCTCACTCGGCTCAAACTCT-3’
Bax	5’-CCGGCGAATTGGAGATGAACT-3’	5’-CCAGCCCATGATGGTTCTGAT-3’
Cd79b	5’-TGTTGGAATCTGCAAATGGA-3’	5’-TAGGCTTTGGGTGATCCTTG-3’
Gapdh	5’-AGGTCGGTGTGAACGGATTTG-3’	5’-TGTAGACCATGTAGTTGAGGTCA-3’
Genotyping		
Sharpin^{+}	5’-TTAGGCACCGAGCCTGGGG-3’	5’-TCGACCAGGTGGCCCGGACATATT-3’
Sharpin^{cpdm}	5’-TTAGGCACCGAGCCTGGGC-3’	5’-TCGACCAGGTGGCCCGGACATATT-3’
Igh^{+}	5’-CCGTCTAGCTTGAGCTATTAGG-3’	5’-GAAGAGGACGATGAAGGTGG-3’
Igh$^{\mu MT}$	5’-CCGTCTAGCTTGAGCTATTAGG-3’	5’-TTGTGCCCAGTCATAGCCGAAT-3’
$V_{186.2}$ amplicon		
$V_{186.2}DJ_{H}$-Cμ	5’-CATGCTCTTCTTGGCAGCAACAGC-3’	5’-CCTGTCCTCAGTGTTGGGAA-3’
$V_{186.2}DJ_{H}$-Cγ1	5’-CATGCTCTTCTTGGCAGCAACAGC-3’	5’-GTGCACACCGCTGGACAGGGATCC-3’
$V_{186.2}DJ_{H}$-Cμ with adaptor R2	5’-CATGCTCTTCTTGGCAGCAACAGC-3’	5’-[GTCTCGTGGGCTCGGAGATGTGTATAAGAGACAG]GAAATGGTGCTGGGCAGGAA-3’
$V_{186.2}DJ_{H}$-Cγ1 with adaptor R2	5’-CATGCTCTTCTTGGCAGCAACAGC-3’	5’-[GTCTCGTGGGGCTC]AGTTTGGGCAGCA-3’
